# Exogenous Ketone Supplements in Athletic Contexts: Past, Present, and Future

**DOI:** 10.1007/s40279-022-01756-2

**Published:** 2022-10-10

**Authors:** Mark Evans, Tyler S. McClure, Andrew P. Koutnik, Brendan Egan

**Affiliations:** 1 School of Health and Human Performance, Dublin City University, Glasnevin, Dublin 9, Ireland; 2 Florida Institute for Human and Machine Cognition, Pensacola, FL USA; 3 National Institute for Cellular Biotechnology, Dublin City University, Dublin, Ireland

## Abstract

The ketone bodies acetoacetate (AcAc) and β-hydroxybutyrate (βHB) have pleiotropic effects in multiple organs including brain, heart, and skeletal muscle by serving as an alternative substrate for energy provision, and by modulating inflammation, oxidative stress, catabolic processes, and gene expression. Of particular relevance to athletes are the metabolic actions of ketone bodies to alter substrate utilisation through attenuating glucose utilisation in peripheral tissues, anti-lipolytic effects on adipose tissue, and attenuation of proteolysis in skeletal muscle. There has been long-standing interest in the development of ingestible forms of ketone bodies that has recently resulted in the commercial availability of exogenous ketone supplements (EKS). These supplements in the form of ketone salts and ketone esters, in addition to ketogenic compounds such as 1,3-butanediol and medium chain triglycerides, facilitate an acute transient increase in circulating AcAc and βHB concentrations, which has been termed ‘acute nutritional ketosis’ or ‘intermittent exogenous ketosis’. Some studies have suggested beneficial effects of EKS to endurance performance, recovery, and overreaching, although many studies have failed to observe benefits of acute nutritional ketosis on performance or recovery. The present review explores the rationale and historical development of EKS, the mechanistic basis for their proposed effects, both positive and negative, and evidence to date for their effects on exercise performance and recovery outcomes before concluding with a discussion of methodological considerations and future directions in this field.

## Key Points

**Table:** 

The ketone bodies acetoacetate (AcAc) and β-hydroxybutyrate (βHB) have wide-ranging metabolic and molecular effects on organs such as the brain, heart, and skeletal muscle, some of which are suggestive of benefits to athletes in terms of performance and recovery.
The recent development and increasing commercial availability of ingestible forms of ketone bodies as exogenous ketone supplements has amplified interest in these products, and resulted in many human exercise studies in the past 5 years.
While there are mechanistic bases for potential beneficial effects of exogenous ketone supplements in various athletic contexts, most studies to date have failed to observe benefits to performance or recovery.
Future research should investigate whether there are other athletic contexts where exogenous ketone supplements are efficacious given the positive, albeit preliminary, data from studies on overreaching, acute hypoxic exposure, and traumatic brain injury.

## Introduction

The ketone bodies (KBs), namely acetoacetate (AcAc) and β-hydroxybutyrate (βHB), are lipid-derived, water-soluble organic compounds produced almost exclusively in the liver, and whose production is amplified most obviously during physiological states characterised by low carbohydrate (CHO) availability (i.e. starvation), prolonged fasting or undertaking ketogenic diets [1–3]. AcAc and βHB have pleiotropic effects in multiple organs including brain, heart, and skeletal muscle by modulating substrate utilisation, inflammation, oxidative stress, catabolic processes, and gene expression [3–5]. In vivo administration of ketogenic compounds was first conducted in patients with paediatric malabsorption disorders (chronic pancreatitis and cystic fibrosis; [6, 7]) even prior to the discovery that these compounds elevate systemic KB concentration [KB] in humans [8]. Development of novel synthetic compounds for the in vivo administration of KBs from exogenous sources has been of interest for ~ 40 years [9–12], initially in the context of parenteral nutrition [13], and more recently with more broad therapeutic applications [14–16]. Early forms included glycerol monobutyrate [9], monoacetoacetin, a monoester of glycerol and AcAc [10, 11], triesters of glycerol and AcAc, and monoesters and triesters of glycerol and βHB [13]. As well as glycerol, R,S-1,3-butanediol (BD) can be esterified to βHB or AcAc, with BD itself in turn elevating [βHB] given its action as a ketogenic precursor [17, 18].

This initial work on administration of KBs from exogenous sources and their potential role in parenteral nutrition, primarily via intravenous infusion, informed the more recent development of ingestible ketone salts and ketone esters, now collectively referred to as exogenous ketone supplements (EKS). βHB is a chiral molecule with two enantiomers, here identified as R- and S-, but also known as D- and L-, respectively. R-βHB is the circulating and primary form of βHB [19, 20], with S-βHB only contributing ~ 3% of [total βHB], even in individuals adhering to a ketogenic diet [21]. Upon entry into peripheral tissues, R-βHB is re-oxidised to AcAc by mitochondrial 3-hydroxybutyrate dehydrogenase (BDH) and then rapidly catabolised to acetyl coenzyme A (CoA) via the ketolytic pathway involving succinyl-CoA:3-oxoacid CoA transferase (OXCT) and acetyl CoA acetyltransferase (ACAT) before entering the tricarboxylic acid (TCA) cycle [1, 22]. S-βHB is biologically present in small quantities, but because it is not a substrate for BDH, S-βHB is not directly metabolised to AcAc and, based on present evidence, makes little direct contribution to energy production [23]. The metabolism of S-βHB remains somewhat poorly described, but is likely to be involved in the hepatic synthesis of free fatty acids (FFA) and sterols, and with a large proportion being converted to R-βHB [24, 25]. However, S-βHB itself does exhibit bioactivity through G-protein coupled receptors [26] and shares similar molecular interactions and intracellular signal transduction cascades with R-βHB [27], and therefore changes in its circulating concentration are likely to have physiological consequences. That said, enantiomer-specific effects have been reported on oxidative phosphorylation in the brain [28], lifespan extension in *Caenorhabditis elegans* [29], glucose utilisation in cardiomyocytes [19], and insulin-stimulated glucose uptake in oxidative skeletal muscle [30], whereby in each instance effects of R-βHB were not similarly observed for S-βHB.

Blood [KB] are typically ≤ 0.1 mM in the postprandial state, and ~ 0.1 to ~ 0.4 mM after an overnight fast [1, 2, 31]. Circulating concentrations may reach ~ 1.0 and ~ 5.0 mM after 24 h and 1 week of fasting, respectively, ~ 0.5 to 3.0 mM on a ketogenic diet, and > 14.0 mM in a state of diabetic ketoacidosis [1, 3, 22, 31]. Hyperketonaemia was accepted originally as circulating [KB] exceeding 0.2 mM [1], whereas circulating [R-βHB] ≥ 0.5 mM has been proposed more recently as an operational definition of ‘nutritional ketosis’ [3, 32]. As noted in later sections (Sects. 2 and 10.1), depending on the method of producing acute nutritional ketosis, there can be divergent responses in [R-βHB], [S-βHB], and [AcAc] such that total [KB] may meaningfully differ from [R-βHB]. In that context, the threshold of [R-βHB] ≥ 0.5 mM is arguably arbitrary. For example, infusion of R-βHB in healthy young men to a concentration of as little as ~ 0.2 to ~ 0.5 mM elicits changes in whole-body metabolism including attenuation of estimated hepatic glucose output and adipose tissue lipolysis, and increases in cerebral R-βHB uptake [33].

**Fig. 1:**
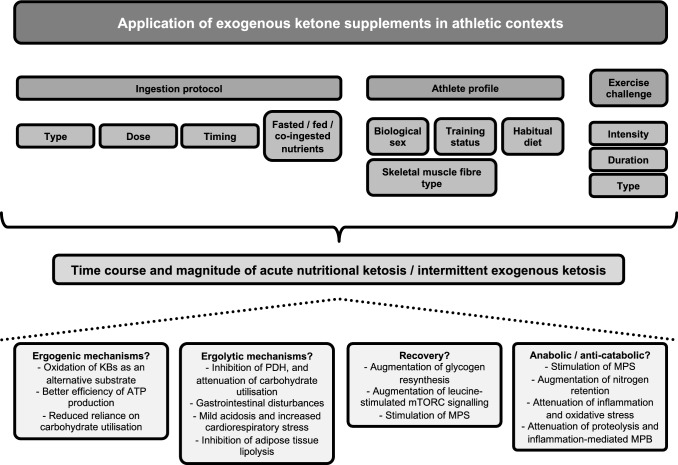
Factors influencing the time course and magnitude of transient changes in circulating concentrations of ketone bodies after acute ingestion of exogenous ketone supplements, and mechanisms of potential benefit and impairment of consequent effects in athletic contexts. *ATP* adenosine triphosphate, *KB* ketone bodies, *MPB* muscle protein breakdown, *MPS* muscle protein synthesis, *mTORC* mechanistic target of rapamycin complex, *PDH* pyruvate dehydrogenase

Ingestion of EKS in athletic contexts has been undertaken primarily with the aim of elevating circulating [R-βHB], and this effect can occur within minutes of ingestion and be maintained for several hours depending on type and dose of EKS, while also being influenced by many factors (Fig. 1), including being fasted or fed, and being at rest or exercising, such that large variations exist in the degree of ketosis (Table 1). Ingestion of EKS, therefore, provides an alternative method to increase [R-βHB], and to a lesser extent [AcAc] [34–39], without injections or intravenous infusions [40], both of which would be impractical or illegal in most athletic contexts.

**Table 1:** Type of exogenous ketone supplements and transient changes in circulating concentrations of ketone bodies after acute ingestion

Supplement type	Brief overview	Dose and changes in circulating [KB]
Medium chain fatty acids (MCFA)/medium chain triglycerides (MCT)	MCTs contain fatty acids 6–12 carbons in length (i.e. MCFAs), and examples include caproic acid (C6), caprylic acid (C8), capric acid (C10), and lauric acid (C12) [318] Compared with long chain fatty acids (LCFAs) being absorbed via the lymphatic system, MCFAs can be absorbed via hepatic portal circulation and enter the hepatic mitochondria without requiring carnitine transport, where they are rapidly metabolised to acetyl CoA, and subsequently to KBs [195] MCTs and MCFAs are therefore considered ketogenic fats as they result in ketogenesis without requiring dietary CHO restriction	Ingestion of MCFA increases circulating [R-βHB] in a dose-dependent manner, with ~ 25 to ~ 30 g and ~ 85 g elevating concentrations to ~ 0.5 mM and ~ 0.9 to ~ 1.5 mM during submaximal exercise, respectively [215, 216, 220]
1,3-butanediol (BD)	BD was developed in 1958 as an alternative source of energy intake for manned space travel and provides ~ 6 kcal.g^−1^ in rodents BD is converted to β-hydroxybutyraldehyde in the liver, and oxidised to R,S-βHB via the action of alcohol and aldehyde dehydrogenase, respectively [18] Ingestion of BD therefore can increase circulating [βHB] in isolation, or can augment increases in circulating [βHB] in response to ketone ester ingestion when present as an esterified component of R-BD R-βHB KME or R,S-BD AcAc KDE with βHB or AcAc, respectively	In the fasted state, ingestion of BD 2 × 0.35 g.kg^−1^ (~ 48 g) elevates whole blood [R-βHB] to ~ 0.5 mM following 85 min steady state at 85% of participants’ VT_2_ and reaching peak concentrations of 1.38 ± 0.35 mM 60 min after a subsequent TT [57] Similarly, in the fasted state, ingestion of BD 0.5 g.kg^−1^ (~ 34 g) ingested alongside CHO (60 g) elevates whole blood [R-βHB] to ~ 0.8 mM following a 1-h pre-load at 75%$\dot{V}{\text{O}}_{2\max }$ and reached peak concentrations of ~ 0.8 mM following a subsequent running TT [56]
Ketone salts (KS)	KS are typically a racemic mixture of R,S-βHB (but can be non-racemic βHB salts or enantiopure R-βHB molecules) bound to a mineral salt or a combination of mineral salts, such as calcium, sodium, or potassium	Several studies have reported circulating [R-βHB] of ~ 0.4 to ~ 1.0 mM in response to ingestion of racemic and non-racemic KS at doses ranging from ~ 10 to ~ 40 g of βHB [21, 34, 36, 45, 46, 51, 52, 62, 85] The majority of commercially available KS being racemic makes them less effective at elevating the R-βHB enantiomer, yet they produce larger (~ twofold greater than R-βHB) and sustained (~ 2.0 mM at 90–120 min) increases in [S-βHB] [34]
Medium chain fatty acids co-ingested with ketone salts (MCFA + KS)	MCFA + KS is most typically a combination of the respective compounds in 1:1 or 2:1 ratios Rodent data suggest this method results in a more sustained induction of nutritional ketosis because KBs are delivered directly in the form of KS, while ketogenesis is stimulated by MCFAs [103] This approach allows for lower dosing of individual components, with lesser potential for side effects from high intake of individual EKS or minerals	Such formulations are available in popular commercialised EKS, but have not been extensively evaluated in human trials Two studies have reported whole blood [R-βHB] of ~ 0.6 mM 60 min after ingestion of a ~ 7 to ~ 9 g R,S-βHB salt with ~ 7 g MCFA [37, 64] Whole blood [R-βHB] was ~ 0.1 mM higher after ingestion of double the above dose [37]
(R)-3-hydroxybutyl (R)-3-hydroxybutyrate (R-BD R-βHB) ketone monoester (KME)	A ketone monoester produced by synthesis of R-β-hydroxybutyrate and R-1,3-butanediol This ketone ester is salt-free, has 99% chiral purity and therefore only provides the R form of βHB [34, 100, 319]	Ingestion in the fasted state produces a rapid and dose-responsive increase in whole blood [R-βHB], e.g. ~ 1.5 mM 20 min after ingestion of 141 mg.kg^−1^ ~ 2.8 mM 60 min after ingestion of 282 mg.kg^−1^ ~ 3.0 mM 30 min after ingestion of 482 mg.kg^−1^ ~ 3.5 mM 10 min after of 573 mg.kg^−1^ and reaching ~ 6.0 and ~ 6.5 mM after 40 and 70 min, respectively [34, 44, 50] Feeding status alters the [R-βHB] response to ingestion, with a prior meal attenuating the increase in circulating [R-βHB] by ~ 30% [34] AcAc kinetics follow a similar time course to R-βHB, but with [R-βHB]:[AcAc] being ~ 6:1 when ingested fasted, and ~ 4:1 when ingested fed at rest [34], and ~ 2:1 during exercise when ingested fed [38]
R,S-1,3-butanediol acetoacetate (R,S-BD AcAc) ketone diester (KDE) ^a^	A ketone diester produced by transesterification of t-butylacetoacetate with R,S-1,3-butanediol [102, 320] This ketone ester is a non-ionised sodium-free and pH-neutral precursor of AcAc	Only one human study has reported circulating [KB] after acute ingestion of R,S-BD AcAc KDE [35] Ingestion of 0.5 g.kg^−1^ (2 × 0.25 g.kg^−1^ 20 min apart) had only modest effects on serum [R-βHB] by increasing to ~ 0.3 to ~ 0.6 mM, although POC measurement of whole blood [R-βHB] was 2- to threefold higher Serum [AcAc] was increased to ~ 0.4 mM
Bis hexanoyl (R)-1,3-butanediol (BH-BD) ketone diester (KDE) ^a^	A ketone diester of hexanoic acid (a 6-carbon ketogenic MCFA also known as caproic acid) and R-1,3-butanediol [39, 105, 106]	Ingestion in the fed state produces a rapid and dose-responsive increase in plasma [R-βHB] [39, 106] e.g ~ 0.4 to ~ 0.8 mM 30–60 min after ingestion of 12.5 g ~ 1.0 to ~ 1.7 mM 60 min after ingestion of 25.0 g

^a^ Not currently (Q2 2022) commercially available

*AcAc* acetoacetate, *βHB* β-hydroxybutyrate, *CHO* carbohydrate, *EKS* exogenous ketone supplements, *KB* ketone bodies, *POC* point-of-care, *TT* time trial, *VT*_*2*_ second ventilatory threshold, $\dot{V}{\text{O}}_{2max }$ maximum rate of oxygen uptake

The consequent acute transient increase in circulating [R-βHB] and [AcAc], which has been termed ‘acute nutritional ketosis’ [41] or ‘intermittent exogenous ketosis’ [42, 43], has been consistently observed to have effects on metabolism both at rest, and during and after exercise [21, 34–38, 42, 44–87]. These effects, combined with interest in KBs as an alternative substrate in the failing heart [88] or in the aging brain [89], have therefore led to considerable interest in EKS as beneficial agents in athletic performance, recovery, and beyond [5, 22, 90–97]. Relatedly, the global market for EKS has grown since becoming commercially available in the latter half of the last decade and is projected to reach ∼ USD$650 million by 2027, with a compound annual growth rate of 5.1% during this period and much of the market currently based in the USA and Asia–Pacific regions [98].

The broad category encompassed by EKS currently includes ketogenic precursors such as BD and medium chain fatty acids (MCFA) and triglycerides (MCT), isolated KBs in the form of R-βHB and R,S-βHB ketone salts (KS), and ketone esters (described in more detail in Table 1). Ketone esters have been prominent in the exercise science literature [35, 38, 42, 44, 47–49, 54, 58–60, 66–68, 70–72, 78, 81, 86, 87, 99], and include the R-3-hydroxybutyl R-3-hydroxybutyrate (R-BD R-βHB) ketone monoester (KME) [34, 44, 100], originally developed to improve the physical and cognitive performance in warfighters [101], and the R,S-1,3-butanediol acetoacetate (R,S-BD AcAc) ketone diester (KDE) [35, 102, 103]. Other ketone esters that have been reported in the peer-reviewed literature to date include a compound of βHB and the short chain fatty acid butyrate (βHB-BA) [104], and a diester of hexanoic acid (a ketogenic MCFA) and R-1,3 butanediol (BH-BD) [39, 105, 106]. Given the numerous possible combinations of AcAc and βHB with ketogenic precursors (including BD, MCFAs, glycerol, and ketogenic amino acids), it is likely that additional forms of EKS will be developed in the future.

In the time since 2016 when the first peer-reviewed article detailing the effects of acute ingestion of EKS in humans on exercise metabolism and endurance performance was published [44], there has been a dramatic increase in the number of articles investigating the effects in humans of acute ingestion of EKS of various types on exercise metabolism, physical and cognitive performance, and recovery from exercise [21, 35–38, 42, 44–49, 51, 52, 54, 56–62, 64, 66–73, 78, 81, 82, 84–86], and in other studies investigating short-term (~ 10 d to 6 weeks) daily consumption [74, 83, 87, 99, 107–110]. Therefore, this review provides an update on investigations into the effects of EKS on exercise performance and recovery outcomes relevant to athletic performance, as well as discussion of methodological considerations and future directions in this field. We have previously reviewed the metabolism of KBs during exercise under conditions of exogenous and endogenous origin [22], the physiological basis for the potential application of EKS for athletic performance [22], and convergence and divergence between nutritional ketosis achieved by dietary manipulation compared with acute ingestion of EKS [3], whereas a detailed history of the development of EKS [111] and the physiology of ketogenesis, ketolysis, and broader metabolic effects of KBs are also reviewed comprehensively elsewhere [1–5, 22, 31, 93].

## Measurement of Circulating Concentrations of Ketone Bodies

At tolerable doses in humans, acute ingestion of MCTs, R,S-BD, KS, and R,S-BD AcAc KDE typically elevate [R-βHB] by ~ 0.3 to 1.0 mM above resting concentrations, whereas acute ingestion of R-BD R-βHB KME typically elevates [R-βHB] in the range of ~ 3 to 6 mM, and concentrations during exercise in the ~ 1.5 to 4.0 mM range (Table 1). These differences between types of EKS are salient because the circulating [R-βHB] is likely to be an important determinant of the metabolic consequences of acute nutritional ketosis. For example, R-βHB is an endogenous ligand of the GPR109A receptor (also known as HCAR2) [5], and has a half-maximal effective concentration (EC_50_) of ~ 0.8 mM in adipocytes [26]. In HEK293 cells, R-βHB treatment increased histone acetylation consequent to inhibition of histone deacetylases (HDACs) 1, 3, and 4 in a dose-dependent manner beginning at 1 mM [112]. Half-maximal inhibitory concentrations (IC_50_) were 5.3 mM, 2.4 mM, and 4.5 mM for HDAC1, HDAC3, and HDAC4, respectively [112].

Therefore, we and others have proposed that concentration-dependent effects of R-βHB and AcAc are likely to exist such that there may be a threshold that acute nutritional ketosis must exceed before effects on skeletal muscle metabolism and exercise performance are observed [3, 22, 91, 93, 94, 96]. Additionally, because the various types of EKS vary in their effectiveness to elevate [R-βHB] (Table 1), we contend that the accurate measurement of [R-βHB] is an important consideration in human studies, especially during mild ketosis, that is, in the ~ 0.3 to 1.0 mM range. Many studies on acute ingestion of EKS have used handheld point-of-care (POC) devices to measure whole blood [R-βHB] [34, 37, 42, 45, 46, 50, 51, 53, 55–59, 61, 62, 64, 67–72, 78, 81, 83–87, 107]. These POC devices were originally developed to monitor and manage ketoacidosis in clinical populations [113–115]. Recently, these devices have increased in popularity as a means to monitor adherence to a ketogenic diet, and use ketone testing strips to provide rapid (< 10 s) feedback to users on their blood [R-βHB]. Indeed, the demand for monitoring KBs in diabetic ketoacidosis, and non-diabetic states of ketosis, has stimulated the development of novel technologies for continuous monitoring of circulating [KB] through sampling of the subcutaneous interstitial fluid [116–119].

POC devices typically use a BDH enzyme-based amperometric strip to establish whole blood [R-βHB] [113–115]. R-βHB is preferred to AcAc for measuring circulating [KB] as an indicator of ketosis, and therefore the use of POC is preferable to urinary ketone measurement due to the inability of nitroprusside in the urinary sticks to detect βHB [114], especially during mild ketosis [120]. Other studies have used laboratory methods, including reagent and colorimetric kits for measurement of plasma or serum [R-βHB] from venous blood samples [35, 36, 44, 47, 49, 54], whereas S-βHB has been determined alongside R-βHB using gas chromatography–mass spectrometry with a chiral column [34], hydrophilic interaction liquid chromatography (HILIC) coupled to electrospray tandem mass spectrometry [65], and the combination of ultraperformance liquid chromatography and electrospray ionisation mass spectrometry [21].

The measurement of S-βHB is not frequently undertaken in studies of EKS, but is worthwhile where possible because of the potential for racemic, non-racemic, and enantiopure versions of βHB salts and BD. Many studies that have used POC measurement of [R-βHB] generally do not observe concentrations > 1.0 mM. Yet in studies that have measured [total βHB] [65, 77], or [R-βHB] and [S-βHB] separately [21, 34], after ingestion of either racemic or non-racemic βHB salts, it is clear that [S-βHB] is markedly elevated, and [total βHB] can be ~ 60% to ~ twofold higher than POC measurement of [R-βHB]. Moreover, S-βHB remains elevated in circulation longer than R*-*βHB (8 h vs 2–4 h) [34], and does not decline at the onset of exercise like R*-*βHB [21]. Given the abovementioned differences in R- and S-βHB metabolism and physiological effects, due consideration should be given to the potential differences between racemic, non-racemic, and enantiopure EKS in future studies.

While POC devices generally exhibit a coefficient of variation (CV) of < 10%, and excellent correlation (*R*^2^ ≥ 0.97) with reference laboratory methods (e.g. RANBUT enzymatic reagent, Randox Laboratories, UK) [113–115], there are differences between devices in their bias for over- or under-estimation [121–124]. For example, recovery of βHB assayed by enzymatic technique indicated that the FreeStyle Optium POC device (Abbott Laboratories, UK) overestimates [R-βHB], whereas the StatStrip POC device (Nova Biomedical, USA) underestimated R-βHB [114]. Of particular relevance to exercise physiology is that the haematocrit value may interfere with R-βHB measurement in some POC devices. For example, blood samples adjusted to range from 24 to 66% in haematocrit exhibited up to a 300% difference in [R-βHB] from the lowest to the highest haematocrit values when measured by FreeStyle Optium but not when measured by StatStrip [113]. In addition, unpublished data from our group have observed that modulating pH results in changes in the measured [R-βHB] using various POC devices (Koutnik & Poff, unpublished), which is notable considering that shifts in pH commonly occur with ingestion of EKS, that is, lower pH with R-BD R-βHB KME, and higher pH with KS [34, 58, 72].

Moreover, in participants fed coffee/cream and 30–50 g of MCFAs, whole blood [R-βHB] was overestimated in capillary samples compared with venous samples tested on the same StatStrip Xpress POC device, and when both were compared with a laboratory method (RANBUT) [123]. Whole blood capillary samples had measured [R-βHB] to be closer to that of total [KB], whereas measured [R-βHB] in venous samples compared more favourably to the laboratory method [123]. Most samples in that study had [R-βHB] of < 1 mM, and because of the differences between measurement methods, revised cut-offs for ketosis were then suggested as 0.3–0.5 mM for plasma samples measured by laboratory assays, 0.3–0.5 mM for venous samples measured by POC, and 0.5–0.8 mM for capillary samples measured by POC devices [123]. Overestimation of [R-βHB] in capillary compared with serum samples was also observed in samples taken prior to and during exercise after ingestion of EKS [35]. [R-βHB] in capillary samples was higher by ~ 0.3 to ~ 0.8 mM during and after exercise when POC (Freestyle Optium Neo) was compared with a colorimetric assay (βHB Assay Kit, Sigma Aldrich, Australia) that measured serum [R-βHB] to be ~ 0.3 to 0.5 mM throughout [35]. Conversely, after ingestion of 600 mg.kg^−1^ of R-BD R-βHB KME when plasma or serum [R-βHB] ranged from ~ 3.4 to ~ 4.4 mM by colorimetric βHB assay, [R-βHB] in capillary samples measured by Freestyle Optium Neo was similar at ~ 3.6 to ~ 4.0 mM [38]. These data suggest that the problem of overestimation of [R-βHB] in capillary samples may be greater at lower concentrations.

In summary, increases in [R-βHB] via EKS ingestion must be critically evaluated based on the measurement method, and the make and model of the testing device in the case of POC, in addition to any confounding factors that may influence the performance of those methods. Hereafter, values reported after measurement by POC are referred to as whole blood [R-βHB], and by laboratory methods as plasma or serum [R-βHB] as appropriate.

## Potential Mechanisms of Action for Exogenous Ketone Supplements as Ergogenic Aids

The main physiological role of the amplification of ketogenesis during low CHO availability is for KBs to replace glucose as the primary source of fuel for the brain, and to a lesser extent provide an additional substrate for other peripheral tissues such as cardiac and skeletal muscle [1, 2, 5]. Elevation of circulating [R-βHB] via infusion as the exogenous means also exerts a range of metabolic actions [40], including attenuating glucose output by the liver [33, 125–127] and glucose utilisation in brain and skeletal muscle [33, 128, 129], lowering circulating [FFA] [33, 127, 130–135] (likely through anti-lipolytic effects on adipose tissue [26]), and attenuating proteolysis and stimulating muscle protein synthesis in skeletal muscle [126, 133, 136–139]. Specific to exercise performance are at least three somewhat inter-related metabolic consequences of acute nutritional ketosis that could potentially result in EKS acting as ergogenic aids (Fig. 1), namely oxidation of KBs as an alternative substrate, better efficiency of adenosine triphosphate (ATP) production when KBs are used as a substrate, and an effect of KBs to elicit reduced reliance on CHO utilisation during exercise.

### Oxidation of KBs as an Alternative Substrate

A number of studies in humans from the late 1960s–1980s using infusions of KBs and/or fasting of various durations [140–147] have made major contributions to the understanding of KB metabolism and substrate utilisation during exercise, as reviewed in detail elsewhere [22, 31]. Briefly, disposal of KBs into skeletal muscle is increased as much as fivefold during exercise in the fasted state, and is reflected by a decrease in circulating [KB] at the onset of exercise [31]. This decrease largely reflects R-βHB being the primary KB extracted from circulation, whereas a net production of AcAc in skeletal muscle has also been observed [31]. These studies were almost exclusively performed in the fasted state, including with prolonged elevations of [KB], which is in contrast to the acute transient increase achieved by EKS and the fact that most competitive athletic performance takes place in the fed state [148]. Interestingly, the metabolic clearance of KBs is enhanced with insulin infusion, at least in the resting state [149], which suggests that co-ingestion of EKS with CHO could potentially augment the oxidation of KBs. Alternatively, because KBs are unlikely to be preferentially used over glucose in skeletal muscle [150, 151], co-ingestion could potentially attenuate the oxidation of KBs.

Traditional stoichiometric equations used to calculate substrate utilisation from the respiratory exchange ratio (RER), oxygen consumption, and carbon dioxide production provide data on absolute oxidation rates (g.min^−1^) and percentage contribution to energy provision for CHO and fat, but assume negligible contributions from other substrates including amino acids, KBs, and lactate [152–154]. During acute nutritional ketosis, careful interpretation of RER during exercise is needed because the stoichiometry of AcAc, the final step in KB oxidation, is 1.00 (i.e. similar to that of CHO), whereas the equivalent value for βHB is 0.89 [152]. However, estimation of oxidation rates of KBs is possible using indirect methods if values for the volume distribution of KBs (i.e. total amount of KBs in the body divided by plasma [KB]), and uptake of KBs into skeletal muscle are known [152].

The first study to estimate the oxidation of KBs after acute ingestion of EKS using these methods employed an analysis of the contribution of βHB to total oxygen consumption in trained athletes during 45 min of cycling at 40% and 75% of maximal power output (*W*_max_) [44]. Ingestion of R-BD R-βHB KME (573 mg.kg^−1^) increased [R-βHB] to ~ 3 mM at the start of exercise, where it remained throughout the 40%*W*_max_ trial, but declined by ~ 1.1 mM during the 75%W_max_ trial. Rates of R-βHB oxidation were estimated to account for ~ 18% and ~ 16% of total oxygen consumption (i.e. energy provision) during the steady-state exercise at 40% and 75%*W*_max_, respectively, with oxidation rates increasing from ~ 0.35 g.min^−1^ at the lower intensity to ~ 0.5 g.min^−1^ at the higher intensity [44]. These percentage contributions and oxidation rates were several-fold higher than had been reported in earlier studies using labelled tracers and the measurement of metabolic clearance rate [31]. This discordance may have been an artefact of the necessity for several assumptions to be made in order to calculate substrate oxidation from gas exchange data using traditional stoichiometric equations that are otherwise unsuitable for use in ketogenic or ketotic states [152, 153]. For example, uptake of KBs into skeletal muscle following EKS ingestion was estimated using the difference between incremental area-under-the-curve of blood [R-βHB] between resting and exercising conditions [44], but such a method does not account for how much of this difference between conditions would be explained by KBs being stored in the form of D-3-hydroxybutyrylcarnitine (also known as ketocarnitine) [155], or lost in the breath and urine [1].

Another potential site of disposal of KBs that is unaccounted for by this method is the utilisation of KBs by the heart. R-βHB can become a major contributor to energy metabolism in cardiac muscle [156], especially so when circulating concentrations are increased [157–160], as would be achieved by acute nutritional ketosis. To our knowledge, myocardial utilisation of KBs during exercise has not been described, but has recently been suggested to be an area of relevant interest in the context of EKS [161, 162]. Shifts in myocardial substrate utilisation analogous to skeletal muscle do occur with the onset of increased contractile activity and increasing exercise intensity and duration [163–168]. Yet, in principle, KBs can alter substrate utilisation in the heart, at least at rest, by reducing reliance on glucose metabolism via product inhibition of key glycolytic and mitochondrial enzymes [169–173] (discussed further in Sect. 4.1). Therefore, whether cardiac muscle is an important site of KB utilisation during exercise, and whether this utilisation is altered by acute ingestion of EKS are intriguing unanswered questions.

Subsequent investigations by the same research group have used ^13^C-labelled R-βHB for a more accurate determination of rates of R-βHB oxidation via breath analysis [67, 68]. These studies in trained endurance athletes with various manipulations of [KB], exercise intensity, and circulating and intramuscular substrate availability, have reported rates of R-βHB oxidation increasing by ~ fivefold to ~ tenfold above rest during aerobic exercise, and absolute values of ~ 0.06 to ~ 0.10 g.min^−1^ [67], and ~ 0.2 to ~ 0.3 g.min^−1^ [68]. However, the approximately threefold difference between the two studies remains unexplained at present. The respective studies estimated the contribution of R-βHB oxidation to energy provision to average ~ 2.5 to ~ 4.5% [67] and ~ 7.4 to ~ 8.4% [68]. These observations are more consistent with values of ~ 2 to ~ 10% reported in the early infusion and prolonged fasting studies [22, 31]. Therefore, KBs make only a minor contribution as a direct source of ATP provision in skeletal muscle during exercise [22, 31], even when [R-βHB] is acutely elevated in the range of ~ 1.7 to ~ 4.5 mM by ingestion of EKS [67, 68]. That there is only this minor contribution is unsurprising given recent in vitro data from permeabilised muscle fibres and isolated mitochondria from skeletal muscle demonstrating that KBs make a minimal contribution to mitochondrial respiration, particularly when other substrates (e.g. pyruvate) are readily available [150, 151].

An important consideration for the oxidation of KBs as an alternative substrate is the expression and activities of enzymes of ketolysis in skeletal muscle, and specifically the effect of exercise training. Adaptations that contribute towards maximising delivery and utilisation of circulating substrates and changes in substrate utilisation patterns during exercise are well established in response to exercise training [174]. As we have reviewed previously [22], from the limited data from humans and rodents, the general pattern in endurance-trained skeletal muscle is for greater capacity for uptake of KBs [175–177], increased activities of the ketolytic enzymes BDH, OXCT, and ACAT [178–181], and higher ex vivo rates of βHB and AcAc oxidation [179, 182]. Similarly, in relation to skeletal muscle fibre types, enzymatic activities of BDH, OXCT, and ACAT are all highest in type I muscle fibres, intermediate in type IIA muscle fibres, and lowest in type IIB fibres of rats [180]. Therefore, we have previously proposed the uptake and utilisation of KBs in skeletal muscle is likely to be greatest in those individuals that are highly endurance-trained with a high proportion of type I muscle fibres and a high oxidative capacity in skeletal muscle [22], a hypothesis for which there is now some preliminary evidence in humans [67].

### Better Efficiency of ATP Production When KBs are Used as a Substrate

Despite the evidently minor contribution of R-βHB to energy provision during exercise, an important question is whether KBs provide a more *efficient* source of ATP provision. Improved energetic efficiency has been proposed as a potential benefit of acute nutritional ketosis [15, 183], and one which may in turn confer an ergogenic benefit [41]. A thermodynamic advantage of R-βHB as a substrate compared with glucose and/or pyruvate is based on the calculation that the available free energy to perform work (Gibbs’ free energy of ATP hydrolysis; ΔG′_ATP_) is greater with R-βHB [15, 41], and that R-βHB produces a higher energy yield per carbon unit, that is, expressed either as kJ/C_2_ or ATP/C_2_ [15, 41, 184]. However, long-chain fatty acids such as a palmitate have higher values for kJ per C_2_ and ATP per C_2_ than R-βHB [15, 41, 184]. An alternative consideration is the P/O ratio, which represents metabolic efficiency as the ATP produced per oxygen consumed (ATP.mol^−1^ O_2_), and indeed R-βHB is more efficient than fatty acids by this metric, but glucose is the most efficient of the three substrates [156, 183].

The often-cited example for improved energetic efficiency associated with KBs as a substrate comes from a perfused working rat heart model where adding KBs to the perfusate suppressed glycolytic flux, and increased hydraulic efficiency (expressed as work in J.mol^−1^ of O_2_ consumed) by 28% [185, 186]. The majority of studies around energetic efficiency of KBs examine myocardial substrate metabolism in healthy and failing hearts, and there is considerable debate about whether this increased efficiency would manifest in in vivo contexts given the competition for substrates [156]. One speculation is that increasing the contribution of KB oxidation to ATP provision should decrease glucose oxidation and potentially decrease cardiac efficiency [156]. However, at present, available data in a working mouse heart model [160] and healthy and failing hearts in humans [187] suggest that acute induction of ketosis can increase the contribution of KBs to energy provision and increase cardiac output and myocardial oxygen consumption, but these effects do not include an increase in cardiac efficiency [156, 160, 187].

The analogous change in skeletal muscle (i.e. improved muscular efficiency) would manifest as a higher power output for the same oxygen consumption, or lower oxygen consumption for the same power output, during exercise performed in acute nutritional ketosis. A salient point is that compared with the abovementioned minor contribution of KBs as a substrate in skeletal muscle, KBs make a much greater contribution to myocardial ATP production in the non-ketotic state (~ 15%) and with elevations in [R-βHB] (~ 25 to 70% at ~ 0.6 to ~ 2.0 mM) [156]. Therefore, it is questionable as to whether a small change in contribution of KBs to energy provision in skeletal muscle during exercise would have a meaningful effect on muscular efficiency. However, an ~ 7% improvement in muscular efficiency (measured as delta efficiency) was recently observed in a study of trained endurance athletes performing cycling exercise at increments of 25%, 50%, and 75%*W*_max_ when [R-βHB] was acutely elevated to ~ 2 mM [67]. This improvement was driven by minor reductions in oxygen consumption and carbon dioxide production at 50% and 75%*W*_max_ compared with the fasting control condition. In contrast are observations of no differences in steady-state oxygen uptake ($\dot{V}{\text{O}}_{2}$) or exercise efficiency compared with control conditions with ingestion of R,S-βHB salts [36, 61], or R-BD R-βHB KME [54, 78], whereas in some cases V̇O_2_ has been observed to be increased [38, 70]. One notable methodological difference between those studies [38, 54, 70, 78] and the observation of improved delta efficiency [67] is that the latter provided R-BD R-βHB KME alone, rather than co-ingestion with CHO, so it remains to be seen whether substrate availability explains these divergent observations.

### Effect of KBs to Elicit Reduced Reliance on Carbohydrate Utilisation During Exercise

Like myocardial energetics, the proportion of contributions from substrates in skeletal muscle during exercise is a complex interaction between exercise intensity, extra- and intramuscular substrate availability, and training status [188]. Whether the observed increase in delta efficiency described above reflects the direct, albeit small, contribution of KBs to energy provision, or whether this is a secondary effect of a change in the contributions of CHO and fat resulting in small changes in the rate of energy expenditure during exercise is presently unknown. Paradoxically, acute nutritional ketosis has been observed to increase the contribution of fat to energy provision (predominately in the form of intramuscular triglyceride) during moderate intensity exercise [44], which, because of the lower P/O ratio of fatty acids compared with glucose, would be expected to *reduce* rather than increase measures of muscular efficiency.

Regardless of whether there is an effect on muscular efficiency, the potential effect of acute nutritional ketosis to result in shifts in substrate utilisation is another mechanism by which ingestion of EKS could exert an ergogenic effect [22, 93]. The proposed mechanism would be the effect of EKS to elicit reduced reliance on CHO utilisation during exercise, an effect also termed ‘glycogen sparing’. Given that performance in many field-based team and endurance sports is CHO-dependent [189–191], athletic endeavours where performance is limited by liver and/or skeletal muscle glycogen stores may benefit from nutrition strategies that ‘spare’ glycogen utilisation [190, 192, 193]. This mechanism of benefit has been explored in the context of acute interventions such as CHO ingestion prior to and during exercise [193], high-dose (≥ 6 g.kg^−1^) caffeine ingestion [194], MCFA/MCT ingestion [195], chronic daily supplementation with L-carnitine [196], and short, medium, and long term adherence to low-CHO/high-fat or ketogenic diets [197], yet overall the results are equivocal as to whether reduced reliance on CHO utilisation during exercise explains the performance benefits when they have been observed.

Specifically in relation to EKS, an initial study observed attenuated CHO utilisation during 2 h of moderate-intensity cycling exercise (~ 70% $\dot{V}{\text{O}}_{2\max }$) after ingestion of R-BD R-βHB KME with CHO as compared with CHO alone [44]. This effect was evidenced by an attenuated rise in blood [lactate], reduced concentrations in skeletal muscle of several intermediaries of glycolytic metabolism, and an attenuation of the decline in skeletal muscle glycogen concentration as assessed by the semi-quantitative method of periodic acid-Schiff (PAS) staining. The attenuated rise in blood [lactate] has been observed in several studies employing ketone esters [35, 44, 49, 58, 67, 72, 86], although not all studies [38, 42, 54, 70], and is generally interpreted as indicative of altered CHO utilisation. However, a subsequent study observed no effect of ingestion of R-BD R-βHB KME on muscle glycogen utilisation during 3 h of intermittent-intensity cycling exercise, or during a 15-min cycling time trial (TT) that followed [42]. That study differed to the previously mentioned study [44] in several important methodological aspects including the provision of a pre-exercise CHO-rich meal, trials being matched for CHO provision, and measuring mixed muscle glycogen concentrations with a quantitative enzymatic assay. Those authors [42] suggest that differences between the two studies are explained largely by the former study [44] being suboptimal in the provision of CHO prior to and during exercise, which contributed to the attenuated utilisation of CHO in skeletal muscle. Further studies under various nutrition manipulations coupled with muscle biopsy analysis will be required to address this question of whether intramuscular substrate utilisation is impacted by EKS.

## Potential Mechanisms of Acute Ingestion of Exogenous Ketone Supplements Resulting in Adverse, or Ergolytic, Effects

### Inhibition of Pyruvate Dehydrogenase and ‘Impairment’ of Carbohydrate Utilisation

The mechanistic basis for the attenuation of CHO utilisation during exercise, whether by high-dose caffeine, pre-exercise high-fat feeding, adherence to a high-fat and ketogenic diet, or acute nutritional ketosis is largely attributed to the inhibition of the enzymatic activity of key metabolic enzymes including phosphofructokinase (PFK) and pyruvate dehydrogenase (PDH) [22, 93, 194, 197]. KBs are proposed to inhibit glycolysis and increase the conversion of glucose to glycogen as demonstrated in rat skeletal muscle in vitro [198], and a perfused heart model in dogs [199], effects mediated by inhibition of PFK and PDH by increases in NADH:NAD^+^, acetyl-CoA:CoA, and/or citrate as a consequence of metabolism of AcAc in mitochondria [172, 186, 198, 199]. In contrast to a proposed benefit of attenuated CHO utilisation during exercise described in Sect. 3.3, it has been proposed that nutrition strategies that attenuate CHO utilisation could paradoxically also negatively impact performance (i.e. an ergolytic effect) [22, 93, 197]. Colloquially, this has been framed as a question of whether such nutrition strategies ‘spare’ CHO, or ‘impair’ CHO utilisation. Data from studies of low-CHO/high-fat and ketogenic diets often support this contention of an ergolytic effect of attenuated CHO utilisation, especially during high-intensity exercise, and this evidence is extensively reviewed elsewhere [197]. One data point worth highlighting is that impairment of performance may result from attenuated activity of PDH, which was reduced during 20 min of cycling at 70% $\dot{V}{\text{O}}_{2\max }$ and during 1 min of supramaximal cycling at 150%*W*_max_ following a 6-day adaptation period to a high-fat diet [200]. Measurement of enzymatic activities of PFK and PDH during exercise after ingestion of EKS will be required to investigate this mechanism further, but it is notable that PDH activity is reduced in cardiac muscle of rats fed a diet supplemented with R-BD R-βHB KME [201].

To date, two studies have demonstrated impaired performance during high-intensity, short-duration (~ 10 to 30 min) cycling TTs after ingestion of R,S-βHB salts [45] or R-BD R-βHB KME [72], although the mechanism of impaired performance was not established in either study. A third study demonstrating impaired cycling TT performance after R,S-BD AcAc KDE attributed the decrement to severe gastrointestinal (GI) disturbances [35], whereas two other studies where trends for impaired high-intensity, short-duration performance were observed also coincided with increased GI disturbances [38, 49]. As a result, GI disturbances are another important consideration for potential ergolytic effects of EKS.

### Gastrointestinal Disturbances and Symptoms

Broadly speaking, GI disturbances during exercise are proposed as physiological, mechanical, or nutritional in nature, and have the potential to negatively impact performance through distraction, discomfort, and/or the attenuation of substrate delivery from exogenous fuel sources [202]. Some concerns exist around EKS because of incidences of GI symptoms reported in several studies after acute ingestion of EKS at rest [52, 65], or prior to exercise [35, 36, 38, 49, 57, 72, 86], and include flatulence, diarrhoea, cramping, belching, heartburn, nausea, and vomiting.

Greater incidences of GI symptoms with the ingestion of KS compared with control conditions have been observed at rest with ~ 30–58 g of racemic R,S-βHB salts [52], and during exercise with ~ 48 g R,S-BD (2 ×  ~ 24 g) provided prior to and during 85 min of steady-state cycling exercise followed by a 7 kJ.kg^−1^ TT [57], and ~ 36 g of racemic R,S-βHB salts (2 ×  ~ 18 g) in the 60 min prior to 48 min of graded cycling exercise [36]. However, this effect may be dose dependent because smaller doses of KS (~ 11 to 12 g R,S-βHB salts) were not associated with greater incidences of GI symptoms [59, 83], or a habituation effect may reduce the likelihood of GI symptoms [83]. A challenge with currently available racemic KS formulations being 50:50 for R- and S-βHB and delivering a high salt and mineral load is to elevate [R-βHB] to the desired concentrations (~ 1.0–3.0 mM) without a risk of developing GI symptoms. The emergence of newer non-racemic formulations with a greater R-βHB component (i.e. 72:28 for R- and S-βHB [21]) suggests that this limitation may be possible to overcome with further innovation and accompanying dose–response studies.

Greater incidences of GI symptoms with the ingestion of ketone esters compared with control conditions has been observed with ~ 37 g of R,S-BD AcAc KDE ingested in the 60 min prior to a 31.2-km cycling TT [35], ~ 59 g of R-BD R-βHB KME ingested 20 min prior to and during an intermittent running protocol lasting ~ 80 min [49], ~ 32 g (females) and ~ 46 g (males) of R-BD R-βHB KME ingested 30 min prior to a 35-min pre-load at ventilatory threshold (VT) followed by a 3-kJ.kg^−1^ cycling TT [38], ~ 50 g of R-BD R-βHB KME ingested during a warm-up in the hour prior to a 30-min cycling TT [72], and ~ 58 g of R-BD R-βHB KME ingested 25 min prior to and during an intermittent running protocol lasting ~ 100 min [86]. However, there are also several studies where greater incidences of the GI symptoms are not observed [42, 54, 59, 62, 70, 78, 81]. In a comprehensive series of studies, ingestion of R-BD R-βHB KME was observed not to increase GI peak symptom load or frequency during exercise [59]. No difference in GI symptoms were observed compared with a CHO-only condition when 65 g of R-BD R-βHB KME was ingested in three doses prior to and during the first hour of 3-h of intermittent intensity cycling exercise [42, 70]. Those authors have observed increased GI symptoms in another study described above [72], and the notable difference compared with their studies where GI symptoms were absent [42, 70] was that in that study, the doses of EKS were ingested in closer proximity to one another, and in closer proximity to commencing the maximum effort 30-min cycling TT that concluded each study. Similarly, our earlier study when marked GI symptoms were observed [49] was in contrast to our later study that observed no differences in GI symptoms between EKS and placebo conditions when ~ 39 g of R-BD R-βHB KME was split into three doses prior to and during 60 min of steady-state treadmill running followed by a 10-km TT [54]. Moreover, despite another of our studies observing high incidence of GI after ingestion of ~ 36 g of R,S-βHB salts [36], negligible GI symptoms were observed in another study using the same product and dosing strategy [62]. At present, there is no clear pattern of supplement type, dosing strategies, and/or exercise type, intensity, or duration that is definitively associated with greater incidence of GI symptoms with acute ingestion of EKS, but a tentative recommendation is that it would be prudent to avoid large doses (> 30 g) in a single bolus in close proximity to high-intensity exercise.

### Mild Acidosis and Increased Cardiorespiratory Stress During Exercise Associated With Acute Nutritional Ketosis

βHB and AcAc are weak organic acids. Unsurprisingly, ingestion of EKS in the form of ketone esters can produce a mild metabolic acidosis evidenced by a decrease in blood pH (~ 0.05 to 0.10) at rest [34] and during exercise [58, 70, 71]. The rapid influx of KBs into circulation creates challenges in maintaining a normal acid–base balance leading to decreases in circulating [bicarbonate] [34, 58, 69]. In most performance contexts, an increased H^+^ load prior to and during exercise is suboptimal for performance as it would limit overall buffering capacity needed to combat the increased H^+^ load during high-intensity exercise [203]. Mild acidosis is therefore associated with ergolytic effects on exercise performance [204], whereas increasing buffering capacity through acute ingestion (e.g. sodium bicarbonate; BIC) or chronic supplementation (e.g. β-alanine) is often ergogenic [205, 206].

An increase in minute ventilation has been observed during 30 min of steady-state cycling exercise at VT [38], 3 h of intermittent intensity cycling exercise [70], and short-duration incremental cycling exercise [58], after ingestion of R-BD R-βHB KME ([R-βHB] ~ 2 to ~ 4 mM). In a related analysis of the latter study [58], subjective reporting of anxiety of breathing and intensity of leg discomfort were higher during acute nutritional ketosis [60]. Mixed effects modelling revealed that pH and [R-βHB] were predictors of these responses, but whole-body ratings of perceived exertion (RPE) were not higher during acute nutritional ketosis despite the localised perceptual effects [60]. This lack of effect on RPE is consistent with most studies of acute ingestion of EKS observing no differences in RPE compared with control conditions. When there has been higher RPE observed in such studies [38, 42, 81], outcomes of the performance tests were not negatively impacted. One notable observation was across a cohort of *n* = 19 males and females; higher RPE compared with the control condition was associated (*r* = 0.64; *p* < 0.01) with reduced 3-kJ.kg^−1^ cycling TT performance after ingestion of R-BD R-βHB KME [38].

Although the mild acidosis in blood associated with ingestion of R-BD R-βHB KME has therefore not been definitively associated with impaired exercise performance, co-administration of BIC has recently been investigated as a strategy to attenuate this acidosis and improve performance [70–72]. In one study of a 15-min cycling TT performed following 3-h of intermittent-intensity cycling exercise, while neither BIC nor R-BD R-βHB KME improved TT performance, KME + BIC improved average power output in the TT by ~ 5% [70]. Another avenue of research is whether declines in pH elicited by KME could have performance benefits in extreme environments such as hypoxia and voluntary hypoventilation [69, 71, 76]. Pre-exercise R-BD R-βHB KME ingestion elicits a range of related effects including increasing the ventilation rate during exercise, lowering PCO_2_ (~ 5–10 mmHg), and shifting the oxygen saturation curve to the left, leading to an ~ 3 to ~ 6% advantage in O_2_ saturation, both in the circulation and skeletal muscle [38, 58, 69, 71, 76]. Thus, rather than being ergolytic, in contexts such as hypoxia and altitude the mild acidosis induced by R-BD R-βHB KME ingestion may be an ergogenic effect of EKS, and this is explored further in Sect. 10.

### Metabolic Consequences of Inhibition of Adipose Tissue Lipolysis

A final consideration of potential for ergolytic effects of EKS is the metabolic consequences of inhibition of adipose tissue lipolysis by KBs [26], given that adipose tissue lipolysis is an important contributor to circulating [FFA] during exercise, and therefore the contribution of fat utilisation to energy provision during long duration, submaximal exercise [174]. The inhibition of lipolysis via nicotinic acid has been observed to impair cycling TT performance in long (120 min), but not shorter (60 min and 90 min) duration efforts [207]. Thus, even in efforts with high CHO dependence (~ 80 to 95% of energy provision), inhibition of lipolysis may impair endurance performance, particularly in long duration activities.

To that point, acute nutritional ketosis achieved by either AcAc infusion [147], or the ingestion of R-BD R-βHB KME or R,S-BD AcAc KDE [35, 42, 44, 58, 67, 72] results in lower circulating [FFA] during exercise than non-ketotic conditions. Attenuation of FFA availability during exercise, and thereby reducing the amount of circulating lipid-derived substrates available to the exercising muscle, typically results in increased intramuscular triglyceride (IMTG) utilisation, and increased glycogenolysis and/or glucose utilisation [208–213]. In contrast, however, is that with ingestion of R-BD R-βHB KME, IMTG utilisation was increased but glycogenolysis was attenuated during 2 h of steady-state moderate intensity (~ 70% $\dot{V}{\text{O}}_{2\max }$) cycling exercise [44], or both were unchanged after 3 h of intermittent cycling exercise and/or a 15-min cycling TT [42].

Clearly, many nodes of metabolic regulation influencing substrate utilisation in exercising skeletal muscle are potentially altered by acute nutritional ketosis, and therefore further investigation is needed to explore the regulation of substrate utilisation during exercise after ingestion of EKS, and whether ergogenic or ergolytic effects are more consistently observed (Fig. 1).

## Effects of Acute Ingestion of Ketogenic Precursors in the Form of Medium-Chain Triglycerides (MCTs) or 1,3-Butanediol (BD) on Exercise Performance

MCT/MCFA and BD are included under the broad category of EKS by virtue of their effects on increasing [R-βHB] after acute ingestion (Table 1), and are therefore considered to be ketogenic precursors. As a means of producing acute nutritional ketosis, MCT/MCFA or BD can each be ingested alone, or used in combination with KBs, typically in the form of MCFA + KS formulations, or in the case of BD as a backbone in the synthesis of the ketone esters R-BD R-βHB KME or R,S-BD AcAc KDE (Table 1).

### MCTs/Medium Chain Fatty Acids (MCFAs)

Historically, MCTs were investigated as a means to increase circulating [MCFA], rather than circulating [KB], and thereby to provide an additional energy source during exercise when oxidation rates of orally ingested glucose reached a maximum at ~ 1.2 g min^−1^ [214]. MCFA oxidation is increased up to ~ 10% of total energy provision when ingested prior to submaximal exercise [215], and endogenous CHO utilisation is attenuated by MCFA intake alone or in combination with CHO when compared with a CHO beverage with a lower dose of CHO [216, 217]. However, this attenuation is not always observed for lower doses of MCFA (~ 25 to ~ 30 g) [218, 219].The attenuation of endogenous CHO utilisation has been postulated to occur via sparing of muscle glycogen because rates of plasma glucose oxidation were unchanged [214]. Ultimately, the effects of MCFA ingestion on exercise performance are equivocal [216, 217, 220, 221], and have been extensively reviewed elsewhere [195]. A known side effect of high MCT consumption is gastrointestinal discomfort [218, 220, 221], which can be mitigated by a slow, progressive introduction over a 1 to 2 week period [222]. Given the incidence of GI symptoms in exercise trials with high doses of MCFA, and the relative lack of increase in [R-βHB] compared with other EKS, the most recent interest in MCFAs as ketogenic precursors is in combination with KS with the aim of eliciting a higher [R-βHB] response than either compound alone (Sect. 7).

### BD

BD is an FDA-approved organic diol used as a food flavouring solvent, and was originally considered as a potential synthetic food for long-duration space missions [223]. When fed in large amounts (> 20% of energy intake) to dogs it can result in a narcotic effect common to glycols [223]. In one study of exercise performance, when BD was ingested prior to exercise, participants reported feelings of euphoria and dizziness, in addition to low level nausea, belching and burping during exercise which may limit the amount of BD that can be ingested prior to exercise to increase circulating [R-βHB] [57]. To our knowledge, only two studies have investigated the effect of acute pre-exercise ingestion of BD on subsequent endurance performance [56, 57] (Table 2).

**Table 2:** Summary of studies of acute ingestion of various types of exogenous ketone supplements on exercise performance

Study Supplement used	Participant profile	Exercise protocol and performance test	Supplement dose, timing, and conditions	Methodological features	Performance outcome
1,3-butanediol (BD)					
Scott et al. (2019) [56] BD	11 males, healthy runners $\dot{V}{\text{O}}_{2\max }$, 64.2 ± 5.0 mL.kg^−1^.min^−1^	60-min treadmill running (75% $\dot{V}{\text{O}}_{2\max }$) 5-km treadmill-based TT	BD + CHO: 0.5 g.kg^−1^ BD + 60 g CHO PLA: 110 ± 5 g CHO Multiple (50:25:25; − 60 & − 30 min pre-exercise, 60 min into exercise)	Double blinded: Yes Placebo: Isocaloric Blinding success: Unclear Randomised: Yes Crossover: Yes Dietary control: 24-h food diary and repeat Pre-trial meal: Fasted Fuel during: CHO	Time to complete (s): BD + CHO: 1261 ± 96 PLA: 1265 ± 93 ES: 0.04 (trivial)
				Validated exercise test: No Sensitivity of exercise test: Not stated Familiarisation trial: Yes βHB measurement: Direct/laboratory assay	
Shaw et al. (2019) [57] BD	9 male cyclists Hours training per week, 12.3 ± 2.3 h $\dot{V}{\text{O}}_{2\max }$, 63.9 ± 2.5 mL.kg^−1^.min^−1^	85-min cycling (85% VT_2_; 73.0 ± 5.2% $\dot{V}{\text{O}}_{2\max }$) 7 kJ.kg^−1^ TT	BD: 0.7 g.kg^−1^ BD PLA: Non-caloric placebo Split (50:50; − 30 min pre-exercise & 60 min into exercise)	Double blinded: Deemed unlikely Placebo: Non-caloric Blinding success: 0% (taste) Randomised: Yes Crossover: Yes	Time to complete (min): BD: 28.7 ± 3.2 PLA: 28.5 ± 3.6 ES: 0.06 (trivial)
				Dietary control: Dietary plan prescribed 6 g.kg^−1^.d^−1^ prior to trials Pre-trial meal: None Fuel during: None Validated exercise test: No Sensitivity of exercise test: Not stated Familiarisation trial: TT only βHB measurement: POC	
Ketone salts (KS)					
O’Malley et al. (2017) [45] KS (KetoForce; KetoSports) Racemic	10 recreationally active males $\dot{V}{\text{O}}_{2\max }$, 45 ± 10 mL.kg^−1^.min^−1^ Exercise ≥ 3 times per week	3 × 5-min cycling (30%, 60%, 90% VT) 150-kJ cycling TT	KS: 0.3 g.kg^−1^ R,S-βHB PLA: Non-caloric placebo Single (− 30 min pre-exercise)	Double blinded: Yes Placebo: Non-caloric Blinding success: 50%	Time to complete (s): KS: 711 ± 137* PLA: 665 ± 120 ES: 0.36 (small)
				Randomised: Yes Crossover: Yes Dietary control: 24-h food diary and repeat Pre-trial meal: Fasted Fuel during: None Validated exercise test: No Sensitivity of exercise test: CV of 2.6% Familiarisation trial: Yes βHB measurement: POC	
Rodger et al., 2017 [46] KS (KetoForce; KetoSports) Racemic	12 male cyclists Professional, A- or B-grade cyclists $\dot{V}{\text{O}}_{2\max }$, 68.0 ± 6.7 mL.kg^−1^.min^−1^	90-min cycling (80% VT_2_) 4-min maximal performance cycling test	KS: 23.4 g R,S-βHB PLA: Non-caloric placebo Split (50:50; − 20 min pre-exercise, 45 min into exercise)	Double blinded: Yes Placebo: Non-caloric Blinding success: Unclear	
				Randomised: Yes Crossover: Yes Dietary control: 48-h food diary and repeat Pre-trial meal: Fasted Fuel during: None Validated exercise test: Yes Sensitivity of exercise test: CV of 2.7% Familiarisation trial: No βHB measurement: POC	Average power output (W): KS: 364 ± 58 PLA: 355 ± 46 ES: 0.19 (trivial)
Waldman et al. (2018) [51] KS (PerfectKeto) Racemic	15 recreationally active males Meeting ACSM exercise recommendations	4 × 15-s maximal cycling sprints	KS: 11.4 g R,S-βHB PLA: Non-caloric placebo Single (− 30 min pre-exercise)	Double blinded: Yes Placebo: Non-caloric Blinding success: Unclear	
				Randomised: Yes Crossover: Yes Dietary control: None Pre-trial meal: Fasted Fuel during: None Validated exercise test: No Sensitivity of exercise test: Not stated Familiarisation trial: Yes βHB measurement: POC	Average power output (W): KS: 715.4 ± 93.5 PLA: 713.8 ± 92.5 ES: 0.02 (trivial)
Kackley et al. (2020) [21] KS + CAF + AA (KETO//OS MAX CHARGED; Pruvit) Non-racemic (72% R-βHB, 28% S-βHB)	12 keto-naïve (KN) individuals (8 male, 4 female) $\dot{V}{\text{O}}_{{2{\text{peak}}}}$, 41.2 ± 5.1 mL. kg^−1^.min^−1^ 12 keto-adapted^*^ (KA) individuals (9 male, 3 female) ^*^At least 3 months consuming ketogenic diet validated by food diary (CHO < 50 g/d), RER < 0.075, and fasting [R-βHB] > 0.5 mM $\dot{V}{\text{O}}_{{2{\text{peak}}}}$, 40.0 ± 10.5 mL. kg^−1^.min^−1^	Incremental cycling time to exhaustion immediately followed by 30-s Wingate test	KS + CAF + AA: 7.2 g R,S-βHB, 100 mg caffeine, 2.7 g taurine, 2.1 leucine CON: Non-caloric water control Single (− 15 min pre-exercise)	Double blinded: No Placebo: No Blinding success: No blinding Randomised: Yes Crossover: Yes Dietary control: 24-h food diary and repeat Pre-trial meal: Fasted Fuel during: None Validated exercise test: No Sensitivity of exercise test: CV of 1.2% (own data) Familiarisation trial: No βHB measurement: POC and direct/laboratory assay	Time to exhaustion (s) KS + CAF + AA KN: 1246 ± 265* KA: 1159 ± 417* CON KN: 1156 ± 260 KA: 1068 ± 404 ES: KN: 0.34 (small) KA: 0.22 (small) Average power output (W) KS + CAF + AA KN: 432 ± 45 KA: 397 ± 24 CON KN: 414 ± 42 KA: 394 ± 40 ES: KN: 0.41 (small) KA: 0.10 (trivial)
Clark et al. (2021) [73]^a^ KS (KetoForce; KetoSports) Racemic	9 healthy active males $\dot{V}{\text{O}}_{{2{\text{peak}}}}$, 56.3 ± 2.2 mL.kg^−1^.min^−1^	30-min cycling (60% *W*_max_) 15-min cycling TT	KS: 0.3 g.kg^−1^ R,S-βHB PLA: Taste-, colour- and electrolyte-matched non-caloric control Single (− 30 min pre-exercise)	Double blinded: Unclear Placebo: Not isocaloric Blinding success: 0%; gastrointestinal symptoms Randomised: Unclear; counterbalanced Crossover: Yes Dietary control: 24-h food diary and repeat Pre-trial meal: Fasted Fuel during: None Validated exercise test: No Sensitivity of exercise test: Not stated Familiarisation trial: No βHB measurement: POC	Average power output in TT (W): KS: 185 ± 40.4 PLA: 190 ± 43.5 ES: 0.11 (trivial)
Quinones & Lemon (2022) [85] KS + CAF + AA (KETO//OS-NAT CHARGED; Pruvit) Non-racemic^b^ KS + AA (KETO//OS-NAT; Pruvit) Non-racemic ^b^	13 healthy, young, recreationally active men Exercise ≥ 2 times per week	20-km cycling TT 15-min rest period Wingate test	KS + CAF + AA: 7 g R,S-βHB, 120 mg caffeine, 2.7 g taurine, 2.1 g leucine KS + AA: 7 g R,S-βHB, 2.7 g taurine, 2.1 g leucine PLA: Isocaloric CHO (~ 11 g) Single (− 30 min pre-exercise)	Double blinded: Yes Placebo: Isocaloric CHO Blinding success: Unclear Randomised: Yes Crossover: Yes Dietary control: None Pre-trial meal: Fasted Fuel during: None Validated exercise test: No Sensitivity of exercise test: Not stated Familiarisation trial: Yes βHB measurement: POC	Time to complete TT (min): KS + CAF + AA: 37.80 ± 2.28 KS + AA: 38.75 ± 2.87 PLA: 39.40 ± 3.33 ES vs PLA: KS + CAF + AA: 0.56 (medium) KS + AA: 0.21 (small) Peak power output (W) Wingate: KS + CAF + AA: 1134 ± 137 KS + AA: 1132 ± 128 PLA: 1068 ± 127 ES vs. PLA: KS + CAF + AA: 0.50 (medium) KS + AA: 0.50 (medium)
Jo et al., 2022 [83]^c^ KS (Creative Compounds) Racemic or non-racemic unclear	32 healthy college-aged endurance-trained individuals (16 male, 16 female) Hours running per week, 4.3 ± 1.3 h	2 × 800-m running TTs (non-motorised treadmill) 5-min active recovery in between TTs	KS + CHO: 11.7 g βHB + 20 g CHO PLA: isocaloric CHO (~ 34 g) Single (− 30 min pre-exercise)	Double blinded: Yes Placebo: Isocaloric CHO Blinding success: Unclear Randomised: Yes Crossover: No Dietary control: 3-d diet log Pre-trial meal: Fasted Fuel during: None Validated exercise test: No Sensitivity of exercise test: Not stated Familiarisation trial: Yes βHB measurement: POC	Time to complete (Δs): KS + CHO: TT1: − 2.3 TT2: − 9.9 ΔTTavg: − 6.1* PLA: TT1: 2.8 TT2: 0.4 ΔTTavg: 1.6 ES: TT1: 0.04 (trivial) TT2: 0.16 (trivial) ΔTTavg: 0.12 (trivial)
Qazi et al (2022) [84] KS (KETO//OS MAX; Pruvit) Non-racemic^b^	19 recreationally active individuals (10 male, 9 female)	Wingate test 10-min rest Incremental test to exhaustion (Bruce treadmill protocol for $\dot{V}{\text{O}}_{{2{\text{peak}}}}$)	KS: 7 g R,S-βHB, 2.7 g taurine, 2.1 g leucine PLA: Isocaloric CHO (~ 10 g) Single (− 30 min pre-exercise)	Double blinded: Yes Placebo: Isocaloric CHO; not taste-matched Blinding success: Unclear Randomised: Yes Crossover: Yes Dietary control: Unclear Pre-trial meal: Fasted Fuel during: None Validated exercise test: Yes Sensitivity of exercise test: Not stated Familiarisation trial: Yes βHB measurement: POC	Average power output (W) Wingate: KS: 500.7 ± 146.0 PLA: 490.2 ± 139.0 ES: 0.07 (trivial) $\dot{V}{\text{O}}_{{2{\text{peak}}}}$ (mL.kg^−1^.min^−1^): KS: 40.91 ± 8.04 PLA: 40.07 ± 7.01 ES: 0.11 (trivial)
Medium chain fatty acids co-ingested with ketone salts (MCFA + KS)					
Prins et al. (2020) [64] KS + MCT (KETO//OS; Pruvit) Racemic	10 male recreational runners Running distance per week, 34.6 ± 5.5 km 5 km time < 30 min in last 3 months	5-km treadmill-based TT	MCFA + KS: 300 mg.kg^−1^ (~ 7 to 9 g R,S-βHB) PLA: Non-caloric placebo Single (− 60 min pre-exercise)	Double blinded: Yes Placebo: Non-caloric Blinding success: Unclear Randomised: Yes Crossover: Yes Dietary control: Unclear Pre-trial meal: Unclear, required not to eat for 3 h prior Fuel during: None Validated exercise test: No Sensitivity of exercise test: ICC, 0.870 Familiarisation trial: Yes βHB measurement: POC	Time to complete (s): MCFA + KS: 1430.0 ± 187.7 PLA: 1488.3 ± 243.8 ES: 0.27 (small)
Prins et al. (2020) [37] KS + MCT (KETO//OS; Pruvit) Racemic	13 recreational male distance runners (60.1 ± 5.4 mL.kg^−1^.min^−1^)	5-km treadmill-based TT	KS + MCT: Low dose βHB + MCT (7 g R,S-βHB, 7 g MCT) High dose βHB + MCT (14 g R,S-βHB, 14 g MCT) PLA: Non-caloric flavour matched Single (− 60 min pre-exercise)	Double blinded: Yes Placebo: Non-caloric Blinding success: Unclear Randomised: Yes Crossover: Yes Dietary control: 3-d food diary and repeat Pre-trial meal: Not stated Fuel during: None Validated exercise test: No Sensitivity of exercise test: Not stated Familiarisation trial: Yes βHB measurement: POC	Time to complete (s): Low dose: 1289.0 ± 104.9 High dose: 1307.3 ± 98.8 PLA: 1291.1 ± 77.1 ES vs. PLA: Low dose: 0.02 (trivial) High dose: 0.18 (trivial)
Ketone esters					
Cox et al. (2016) [44] KME (DeltaG; TdeltaS / University of Oxford)	8 endurance athletes (6 male, 2 female) $\dot{V}{\text{O}}_{2\max }$, M: 5.37 ± 0.3 L.min^−1^; F: 3.30 ± 0.1 L.min^−1^	60-min cycling (75% *W*_max_) 30-min cycling TT (max distance)	KME + CHO: 573 mg.kg^−1^ KME (40% EI) + 1.2 g.min^−1^ CHO PLA: Isocaloric CHO as 40% dextrose, 40% fructose, 20% maltodextrin Multiple (50:25:25, − 20 min pre-exercise, 30 & 60 min into exercise)	Double blinded: Unclear Placebo: Isocaloric CHO Blinding success: Unclear Randomised: Yes Crossover: Yes Dietary control: Unclear Pre-trial meal: Fasted Fuel during: CHO Validated exercise test: No Sensitivity of exercise test: Unclear Familiarisation trial: No βHB measurement: Direct/laboratory assay	Distance covered (m): PLA: ~ 20,000 m cycled in PLA KME + CHO: cycled 411 ± 162 m further (mean ± SEM)
Leckey et al. (2017) [35] KDE (Savind / University of South Florida)	10 male cyclists, internationally competitive $\dot{V}{\text{O}}_{2\max }$, 71.4 ± 5.6 mL.kg^−1^.min^−1^	4 × 5-min cycling as standardised warm-up 31.17-km cycling TT	KDE + CHO: 500 mg.kg^−1^ KDE + 250 mL 6% CHO drink PLA: 250 mL 6% CHO drink Split (50:50; − 30 & − 5 min pre-exercise)	Double blinded: Yes Placebo: Not isocaloric Blinding success: 0%, gastrointestinal symptoms Randomised: Yes Crossover: Yes Dietary control: Food provided evening prior to trials, 3 g.kg^−1^ CHO Pre-trial meal: 2 g.kg^−1^ CHO Fuel during: Caffeine and CHO Validated exercise test: No Sensitivity of exercise test: Not stated Familiarisation trial: Yes βHB measurement: POC and direct/laboratory assay AcAc measurement: Direct/laboratory assay	Average power output (W) KDE + CHO: 339 ± 37* PLA: 352 ± 35 ES: 0.42 (small)
Evans & Egan (2018) [49] KME (KE4; KetoneAid)	11 male team sport athletes Actively competing in high intensity field sports $\dot{V}{\text{O}}_{{2{\text{peak}}}}$, 53.9 ± 2.2 mL.kg^−1^.min^−1^	LIST: 5 × 15 intermittent intensity running activity 20-m shuttle run to exhaustion (alternating 55% and 95% of speed at $\dot{V}{\text{O}}_{2\max }$)	KME + CHO: 750 mg.kg^−1^ KME + 1.2 g.min^−1^ CHO PLA: 1.2 g.min^−1^ CHO Multiple (50:25:25; − 30 min pre-exercise, 15 & 30 min into exercise)	Double blinded: Yes Placebo: Not isocaloric Blinding success: 27% Randomised: Yes Crossover: Yes Dietary control: Food provided day prior to trials as 40 kcal.kg^−1^, 60% CHO, 20% fat, 20% protein Pre-trial meal: Food provided day of trials as 3 g.kg^−1^ CHO Fuel during: 6.4% CHO beverage Validated exercise test: Yes Sensitivity of exercise test: Not stated Familiarisation trial: Yes βHB measurement: Direct/laboratory assay	Time to exhaustion (s): KME + CHO: 229 ± 72 PLA: 267 ± 96 ES: 0.45 (small)
Evans et al. (2019) [54] KME (DeltaG; HVMN)	8 middle and long distance (7 male, 1 female) $V{\text{O}}_{{2{\text{peak}}}}$, 62.0 ± 5.6 mL.kg^−1^.min^−1^	60-min treadmill running (65% $\dot{V}{\text{O}}_{2\max }$) 10-km treadmill-based TT	KME + CHO: 573 mg.kg^−1^ KME + 1.0 g.min^−1^ CHO PLA: 1.0 g.min^−1^ CHO Multiple (50:25:25; − 30 min pre-exercise, 20 & 60 min into exercise)	Double blinded: Yes Placebo: Not iso-caloric Blinding success: 75% Randomised: Yes Crossover: Yes Dietary control: Prescribed dietary plan of trials ~ 2800 kcal at 60% CHO (~ 6.2 g.kg^−1^), 20% protein and 20% fat Pre-trial meal: ~ 300–400 kcal as ~ 1.0 g.kg^−1^ CHO 2 h prior to exercise Fuel during: 8.0% CHO beverage Validated exercise test: Yes Sensitivity of exercise test: CV of 1.5% Familiarisation trial: Yes βHB measurement: Direct/laboratory assay	Time to complete (s) KME + CHO: 2402 ± 237 PLA: 2422 ± 246 ES: 0.08 (trivial)
Dearlove et al. (2019) [58] KME (DeltaG; TdeltaS / University of Oxford)	12 healthy athletes (9 male, 3 female) Minimum 6 h endurance training per week $\dot{V}{\text{O}}_{2\max }$, 4.4 ± 0.2 L.min^−1^	Incremental cycling test to exhaustion	KME: 330 mg.kg^−1^ KME PLA: Non-caloric placebo Single (− 60 min pre-exercise)	Double blinded: No, single blinded Placebo: Non-caloric Blinding success: Unclear Randomised: Yes Crossover: Yes Dietary control: None Pre-trial meal: Fasted Fuel during: None Validated exercise test: No Sensitivity of exercise test: Not stated Familiarisation trial: No βHB measurement: POC	Peak power output (W) KME: 393 ± 22 PLA: 389 ± 20 ES: 0.05 (trivial)
Poffé et al. (2020) [42] KME (DeltaG; TdeltaS / University of Oxford)	12 trained male cyclists $\dot{V}{\text{O}}_{2\max }$, 62.4 ± 6.6 mL.kg^−1^.min^−1^	180-min cycling (6 × 30-min blocks at varying intensities) 15-min cycling TT followed by sprint cycle to exhaustion (175% LT W)	KME: 65 g (918 ± 102 mg.kg^−1^ KME) + 60 g.h^−1^ CHO PLA: Taste- and volume-matched CP + 60 g.h^−1^ CHO Multiple (25/20/20 g; − 60 & − 20 min pre-exercise, 30 min into exercise)	Double blinded: Yes Placebo: Not isocaloric Blinding success: 42% successful Randomised: Yes Crossover: Yes Dietary control: Food provided evening prior to trials, high CHO meal (~ 5600 kJ, 69% CHO) Pre-trial meal: Breakfast provided (~ 2600 kJ; 72% CHO) Fuel during: 6% CHO drink and energy bar Validated exercise test: No Sensitivity of exercise test: Not stated Familiarisation trial: Yes βHB measurement: POC	Average power output (W) in TT: KME: 273 ± 38 PLA: 272 ± 37 ES: 0.02 (trivial) Time to exhaustion (s) in sprint: KME: 59 ± 16 PLA: 58 ± 17 ES: 0.06 (trivial)
Poffé et al. (2021) [70]^d^ KME (DeltaG; TdeltaS / University of Oxford)	9 trained male cyclists $\dot{V}{\text{O}}_{2\max }$, 61.0 ± 2.9 mL.kg^−1^.min^−1^	180-min cycling (6 × 30-min blocks at varying intensities) 15-min cycling TT followed by sprint cycle to exhaustion (175% LT W)	KME: 65 g (922 ± 85 mg.kg^−1^) KME + 60 g.h^−1^ CHO PLA: Taste- and volume-matched CP + 60 g.h^−1^ CHO Multiple (25/20/20 g; − 60 & − 20 min pre-exercise, 30 min into exercise)	Double blinded: Yes Placebo: Not isocaloric Blinding success: 6/9 (67%) successful Randomised: Yes Crossover: Yes Dietary control: Food provided evening prior to trials, high CHO meal (~ 5600 kJ, 69% CHO) Pre-trial meal: Breakfast provided (~ 2600 kJ; 72% CHO) Fuel during: 6% CHO drink and one energy bar Validated exercise test: No Sensitivity of exercise test: Not stated Familiarisation trial: Yes βHB measurement: POC	Average power output (W) in TT: KME: ~ 254 PLA: ~ 254 ES: 0.0 (trivial) Time to exhaustion (s) in sprint: KME: 55 ± 19 PLA: 55 ± 21 ES: 0.0 (trivial)
Poffé et al. (2021) [72] KME (DeltaG; TdeltaS / University of Oxford)	12 highly trained, male cyclists $\dot{V}{\text{O}}_{2\max }$, 62.5 ± 5.5 mL.kg^−1^.min^−1^	60-min standardised warm-up 30-min cycling TT followed by sprint cycle to exhaustion (175% LT W)	KME: 50 g KME (726 ± 75 mg.kg^−1^) PLA: Taste- and volume-matched CP Split (50:50; during warm-up as − 30 & − 5 min pre-TT)	Double blinded: Yes Placebo: Not isocaloric Blinding success: No subjects correctly identified trial sequence Randomised: Yes Crossover: Yes Dietary control: Food provided evening prior to trials, high CHO meal (~ 5600 kJ, 69% CHO) Pre-trial meal: Breakfast provided (~ 2600 kJ; 72% CHO) Fuel during: 60 g during warm-up Validated exercise test: No Sensitivity of exercise test: Not stated Familiarisation trial: Yes βHB measurement: POC	Average power output in TT: 3.8 ± 1.5 W lower* in KME than PLA ES: 0.13 (small) Time to exhaustion (s) in sprint not different between KME and PLA
Poffé et al. (2021) [71] ^e^ KME (DeltaG; TdeltaS / University of Oxford)	14 highly trained male cyclists $\dot{V}{\text{O}}_{2\max }$, 64.6 ± 6.6 mL.kg^−1^.min^−1^	180-min cycling (6 × 30-min blocks at varying intensities) 15-min cycling TT followed by sprint cycle to exhaustion (175% LT W) Trials performed in hypobaric altitude Hypobaric altitude began increasing at start of exercise, peaked at 3000 m at 150 min into exercise	KME: 75 g (1019 ± 111 mg.kg^−1^ KME) + 60 g.h^−1^ CHO PLA: Taste- and volume-matched CP + 60 g.h^−1^ CHO Multiple (25/25/25 g; 30, 90 & 150 min into exercise)	Double blinded: Yes Placebo: Not isocaloric Blinding success: No subjects correctly identified trial sequence Randomised: Yes Crossover: Yes Dietary control: Food provided evening prior to trials, high CHO meal (~ 5600 kJ, 69% CHO) Pre-trial meal: Breakfast provided (~ 2600 kJ; 72% CHO) Fuel during: 6% CHO drink and one energy gel Validated exercise test: No Sensitivity of exercise test: Not stated Familiarisation trial: Yes βHB measurement: POC	Average power output (W) in TT: KME: 248 ± 21 PLA: 246 ± 26 ES: 0.08 (trivial) Time to exhaustion (s) in sprint: KME: ~ 52 s PLA: ~ 55 s
McCarthy et al. (2021) [38] KME (DeltaG; HVMN)	19 endurance-trained individuals (10 male, 9 female) $\dot{V}{\text{O}}_{2\max }$, M 61 ± 7 mL.kg^−1^.min^−1^; F 53 ± 6 mL.kg^−1^.min^−1^) Endurance training ≥ 3 times per week	30-min cycling at VT (71 ± 3% $\dot{V}{\text{O}}_{{2{\text{peak}}}}$) followed by 15-min rest prior to a 3-kJ.kg^−1^ cycling TT	KME: 600 mg.kg^−1^ KME + 25 g sports drink powder PLA: Flavour-matched PLA + 25 g sports drink powder Single (− 35 min pre-exercise)	Double blinded: Yes Placebo: Not isocaloric Blinding success: unclear Randomised: Yes Crossover: Yes Dietary control: 24-d diet record and repeat Pre-trial meal: Standardised breakfast of ~ 1 g.kg^−1^ CHO Fuel during: None Validated exercise test: Yes Sensitivity of exercise test: ~ 2.5% CV between trials 2 and 3 Familiarisation trial: Yes βHB measurement: POC and direct/laboratory assay	Average power output (W) [median (IQR)]: KME: 196 (176–295) PLA: 201 (174–279) ES: 0.32 (small) Time to compete (min:s) KME: 16:25 ± 2:50 PLA: 16:06 ± 2:40 ES: 0.31 (small)
Waldman et al. (2022 [81] KME (KE4; KetoneAid)	14 male professional firefighters	Live-burn search and rescue	KME: 500 mg.kg^−1^ KME PLA: Non-caloric taste-matched Single (− 30 min before search and rescue)	Double blinded: Yes Placebo: Non-caloric Blinding success: Yes Randomised: Yes Crossover: Yes Dietary control: 24-d diet record and repeat Pre-trial meal: Fasted Fuel during: None Validated exercise test: No Sensitivity of exercise test: Unclear Familiarisation trial: No βHB measurement: POC	Time to completion (min): KME: 10.6 ± 0.6 PLA: 10.6 ± 0.8 ES: 0.0 (trivial)
Peacock et al. (2022) [86] KME (DeltaG; TdeltaS/University of Oxford)	9 professional male rugby players	Simulated rugby union match-play protocol (BURST; ~ 100 min) with performance outcomes throughout the protocol via high intensity performance test, 15-m sprint times, and sled push test	KME + CHO: 590 mg.kg^−1^ KME + 20 ± 2 g CHO PLA: Tasted-matched PLA + 90 ± 9 g CHO Split (2/3 pre-exercise, 1/3 mid-exercise)	Double blinded: Yes Placebo: Isocaloric Blinding success: Yes Randomised: Yes	Average time to completion in performance test (s): KME + CHO: 15.53 ± 0.52 PLA: 15.86 ± 0.80 ES: 0.48 (medium)
				Crossover: Yes Dietary control: 48-h diet record and repeat Pre-trial meal: Habitual breakfast ~ 165 g CHO	
				Fuel during: CHO drinks Validated exercise test: Yes	
				Sensitivity of exercise test: High intensity performance test CV 1.4%; 15-m sprints CV 1.8%; sled push test CV 0.6% Familiarisation trial: Yes βHB measurement: POC	No differences in 15-m sprint times or sled push test times

Studies of MCFAs or MCTs are not included, but have been reviewed elsewhere [195]

*AA* amino acids, *ACSM* American College of Sports Medicine, *BD* 1,3-butanediol, *BURST* Bath University Rugby Shuttle Test, *CAF* caffeine, *CHO* carbohydrate, *CP* collagen peptides, *CV* coefficient of variation, *EI* energy intake, *ES* effect size, *ICC* intraclass correlation coefficient, *IQR* interquartile range, *KDE* R,S-1,3-butanediol acetoacetate (R,S-BD AcAc) ketone diester, *KME* (R)-3-hydroxybutyl (R)-3-hydroxybutyrate (R-BD R-βHB) ketone monoester, *KS* ketone salts, *LIST* Loughborough Intermittent Shuttle Test, *LT* lactate threshold, *MCFA* medium chain fatty acids, *MCT* medium chain triglycerides, *PLA* placebo condition, *POC* point-of-care, *RER* respiratory exchange ratio, *SD* standard deviation, *SEM* standard error of the mean, *TT* time trial, $\dot{V}{\text{O}}_{2\max }$ maximum rate of oxygen uptake, *VT* ventilatory threshold

*Indicates *p* < 0.05 for EKS compared with placebo or control condition. All data are mean ± SD unless otherwise stated. ES are calculated as Cohen’s *d*

^a^Study also includes a condition of KS with whole-body cooling that is not included in this summary

^b^Assumed to be non-racemic based on the analysis of Kackley et al. [21] for the same product (KETO//OS MAX; Pruvit). Also of note is that earlier versions of this product line were racemic (KETO//OS; Pruvit) [37, 64], whereas most recently KETO//OS-NAT (Pruvit) is purported to contain R-βHB free acid (i.e. the pure enantiomer)

^c^Trial of acute effects of ingestion of KS was preceded by 10 d of daily ingestion of same KS

^d^Study also includes conditions of sodium bicarbonate alone, and sodium bicarbonate plus KME that are not included in this summary

^e^Study also includes conditions of sodium bicarbonate alone, and sodium bicarbonate plus KME that are not included in this summary

The first study employed a 60-min pre-load of submaximal running followed by a 5-km treadmill-based running TT, but observed no differences in TT performance with ingestion of 0.5 g.kg^−1^ BD compared with placebo in an energy-matched CHO-based comparison (BD + CHO: 1261 ± 96 s; CHO: 1265 ± 93 s) [56]. Similarly, there was no difference in performance in a 7-kJ.kg^−1^ cycling TT when 2 × 0.35 g.kg^−1^ BD was co-ingested with 60 g CHO, compared with when 110 ± 5 g CHO was ingested prior to exercise (BD + CHO: 28.7 ± 3.2 min; CHO: 28.5 ± 3.6 min) [57]. In both studies, [R-βHB] averaged < 1 mM throughout the exercise period, and therefore, the low level perceptual and GI symptoms, coupled to this modest effect on circulating [R-βHB], suggest that BD is unlikely to have ergogenic potential in athletes.

## Effects of Acute Ingestion of Ketone Salts on Exercise Performance

The most direct method of exogenously inducing nutritional ketosis would be to ingest isolated KBs. However, R-βHB and AcAc in their free acid form can be unstable, expensive, and ineffective at producing sustained ketosis. Thus, the ketone acids can be buffered with sodium or other electrolytes to enhance efficacy and prevent overload of any single mineral. Currently, most commercially available ingestible KS are a racemic mixture of R-βHB and S-βHB enantiomers of βHB (i.e. R,S- βHB), largely because the synthesis of racemic mixtures is more affordable than the non-racemic mixtures and pure enantiomers.

There are eight studies to date on the effects of acute ingestion of KS on exercise performance [21, 45, 46, 51, 73, 83–85] (Table 2). The earliest of those studies observed *impaired* performance after ingestion of 0.3 g.kg^−1^ of KS (~ 25 g R,S-βHB) 50 min prior to exercise, which increased whole blood [R-βHB] to ~ 0.8 mM, but resulted in lower average power output by 7% (− 16 W) and increased time to completion (KS: 711 ± 137 s; placebo [PLA]: 665 ± 120 s) for a 150-kJ cycling TT (~ 10 km) [45]. Several studies since then have observed no effect on exercise performance [46, 51, 73, 84]. The first of these studies provided two servings of KS providing 11.7 g of R,S-βHB each, the first being at 30 min prior to exercise and the second being 45 min into a 90-min pre-load at 80% VT_2_ [46]. Upon completion of the pre-load, participants performed a 4-min maximal cycling performance test. The dosing described increased whole blood [R-βHB] to 0.6 ± 0.3 mM by the end of the pre-load, but there was no difference between KS and the placebo group for average power output during the performance test (KS: 364 ± 58 W; PLA: 355 ± 46 W) [46]. Similarly, ingestion of a serving of KS providing 11.4 g of R,S-βHB 30 min prior to exercise increased whole blood [R-βHB] to 0.53 ± 0.19 mM, but had no effect on average power output (KS: 715 ± 94 W; PLA: 714 ± 93 W), or peak power output (KS: 969 ± 157 W; PLA: 955 ± 151 W) during 4 × 15 s of maximal sprints on a cycle ergometer [51]. More recently, 0.3 g.kg^−1^ of KS (~ 23 g R,S-βHB) was ingested 30 min prior to commencing a 30-min pre-load at 60% *W*_max_ followed by a 15-min cycling TT [73]. Whole blood [R-βHB] was increased to ~ 0.9 mM 30 min after ingestion but had declined to ~ 0.5 mM prior to the start of the TT, and average power output during the TT was not different between conditions (KS: 185 ± 40 W; PLA: 190 ± 43 W) [73].

An ergogenic effect of acute ingestion of KS on exercise performance has been observed in two studies [21, 85], but is arguably confounded by the fact that the ergogenic effect was observed with a multi-ingredient pre-workout formulation containing 100 mg of caffeine, 2.1 g of L-leucine, and 2.8 g of L-taurine in addition to 7.2 g of non-racemic βHB salts. In the first study, the control condition was plain water, and time-to-exhaustion in an incremental cycling test was increased in participants consuming a mixed diet (+ 8.3%), and in those consuming a ketogenic diet (+ 9.8%) [21]. The plasma [R-βHB] during exercise was ~ 0.5 mM in the mixed diet group and ~ 1.7 mM in the ketogenic diet group [21]. In a follow-up study using the same formulation and dosing strategy, an ergogenic effect was again observed [85], Notably, that study included a caffeine-free version of the multi-ingredient pre-workout KS formulation and an isoenergetic CHO placebo. In the caffeinated formulation, average power output in a 20-km cycling TT was ~ 5.6% and ~ 9.5% greater than the caffeine-free and CHO conditions, respectively, whereas the caffeine-free and CHO conditions were similar [85]. However, in a 30-s Wingate test, both versions of the KS formulation improved peak power output by ~ 6.2% compared with CHO. These data led the authors to suggest that, depending on the performance outcome, the presence of caffeine or taurine, rather than KS per se, is likely to be a strong influence on the ergogenic effects of that formulation [85]. A subsequent study provided the same caffeine-free version of the KS formulation 30 min prior to a Wingate test, which was then followed 10 min later by an incremental cycle test to exhaustion to measure $\dot{V}{\text{O}}_{2\max }$ [84]. Whole blood [R-βHB] peaked at ~ 1.3 mM prior to the Wingate test, and remained elevated at ~ 1.0 and ~ 0.4 mM after the Wingate and incremental tests, respectively, but no effects of KS were observed on Wingate test outcomes (peak power output, average power output, fatigue index) or $\dot{V}{\text{O}}_{2\max }$ compared with isocaloric CHO [84].

Lastly, Jo et al. [83] observed an ergogenic effect of KS with 10 days of daily KS supplementation followed by acute ingestion of KS 30 min prior to a performance test involving 2 × 800-m running TTs on a non-motorised treadmill separated by 5 min’ rest. The KS supplement provided 11.7 g of sodium βHB salts (whether racemic or not was not stated) and 20 g of CHO, and increased whole blood [R-βHB] by ~ 0.4 mM, whereas the control condition was isoenergetic CHO alone. Performance times in the first and second TT separately were not significantly improved from pre- to post-intervention, but the performance time averaged across the two TTs improved by 6.6 ± 8.9 s (~ 2.7%) after KS supplementation [83]. An intriguing question is whether it was employing daily consumption for 10 days that contributed to this ergogenic effect that has been absent in other KS studies, or whether the performance test incorporating repeated ~ 4-min efforts specifically benefited from acute nutritional ketosis.

Ultimately, there is little evidence overall for ergogenic effects of acute ingestion of KS in the form of R,S-βHB salts, albeit all of the performance outcomes have focussed on short duration, high-intensity exercise performance tests, but the data are also consistent in the finding that increases in circulating [R-βHB] are modest (generally < 1.0 mM) under the dosing strategies employed to date.

## Effects of Acute Co-Ingestion of Ketone Salts and MCFA on Exercise Performance

Given the modest effects of both MCFA and KS alone to increase circulating [R-βHB], a possible strategy is to combine the two compounds given their independent mechanisms of increasing [R-βHB] (i.e. as ketogenic precursors and βHB enantiomers, respectively). This co-ingestion strategy was initially observed to augment the circulating [R-βHB] response in a rodent model [103]. To date, two studies have investigated the metabolic and performance effects of simultaneous MCFA and KS ingestion in humans [37, 64]. Both studies employed the same design of ingestion of a commercially available MCFA + KS supplement 60 min prior to performing a 5-km treadmill-based running TT. The MCFA + KS supplement contained ~ 7 to ~ 9 g R,S-βHB and ~ 7 g MCFAs per serving, but whether consumed as a single serving [37, 64] or double serving [37] had no effect on the primary outcome of time to complete the 5-km TT when compared with a flavour-matched non-caloric placebo (Table 2). Whole blood [R-βHB] was increased to ~ 0.6 mM 60 min after ingestion of a single serving [37, 64], and was ~ 0.1 mM higher at the same timepoint after ingestion of the double serving [37]. Of note was that [R-βHB] was reduced to ~ 0.3 mM after the TT performance after ingesting a single serving, but remain elevated to ~ 0.6 mM in the double serving condition. However, there was no effect on blood [glucose], [lactate], heart rate, or RPE between conditions or doses. The authors noted that although TT performance in the first study was not different between MCFA + KS (1430 ± 188 s) and placebo (1488 ± 244 s), eight out of ten participants had improved performance after MCFA + KS ingestion, but this pattern of improvement was not reproduced in the second study. Given the presence of S-βHB enantiomer in racemic KS, and the relatively low dose (~ 14 g) of MCFA, the sustained increase in [R-βHB] in the double serving condition suggests that further investigation with non-racemic or enantiopure βHB salts with doses of MCFA, potentially combined with CHO provision, would be worthwhile.

## Effects of Acute Ingestion of Ketone Esters on Exercise Performance

Ketone esters is a term used to describe R-βHB and AcAc molecules attached either to another ketone body or a ketone body precursor via an ester bond, which is then cleaved by gastric esterases to liberate KBs in their free acid form from a backbone molecule. The choice of backbone molecule can vary, but most often is a ketogenic precursor molecule such as R-BD or R,S-BD [34, 35, 44, 100, 102, 103, 105, 106]. Ketone esters, especially R-BD R-βHB KME, are currently the most potent EKS available in terms of reliably and robustly producing acute nutritional ketosis (Table 1). In the following sections, we specify the dosing strategy for the ketone esters ingested, but additional details on the fuelling strategies before (fasted or fed), and during (e.g. CHO co-ingestion) are described in Table 2.

In a now seminal study, ingestion of 573 mg.kg^−1^ R-BD R-βHB KME increased circulating [R-βHB] to ~ 2.0 to ~ 2.5 mM during exercise, and improved 30-min maximum distance cycling TT performance by ~ 2% (411 ± 162 m; mean ± SEM) following a 1-h pre-load at 75% *W*_max_ [44]. The drinks consumed in that study were isocaloric, meaning performance improved despite providing less CHO throughout the R-BD R-βHB KME condition [44]. Since this publication, several studies of the effects of acute ingestion of ketone esters on exercise performance have been reported [35, 38, 42, 49, 54, 58, 70–72, 78, 81, 86] (Table 2). However, with one exception [86], these studies have failed to observe an ergogenic effect of R-BD R-βHB KME. One study has observed an ergolytic effect of R-BD R-βHB KME [72], whereas the only study to date of R,S-BD AcAc KDE ingestion observed a 2 ± 1% (58.2 s) decrement in 31.2-km TT performance in professional cyclists [35].

In that study, participants ingested a 500-mg.kg^−1^ dose of R,S-BD AcAc KDE as two 250-mg.kg^−1^ boluses at 50 min and 30 min prior to the TT, having consumed a high CHO breakfast (2 g.kg^−1^) on the morning of each trial. Participants also received 200 mg of caffeine, a 6% CHO sports drink and a caffeine gel during the trial to mimic the real-world practice in professional cycling. The plasma [R-βHB] of ~ 0.4 mM in that study [35] was markedly lower than studies that have used R-BD R-βHB KME [38, 42, 49, 54, 58, 70–72, 78, 81, 86]. R,S-BD AcAc KDE ingestion at this dose was, therefore, ineffective at meaningfully increasing [R-βHB], but ultimately the performance decrement was explained by a 3.7% reduction in average power output, and was accompanied by a high prevalence of GI symptoms among participants in the R,S-BD AcAc KDE condition [35].

Our group has since performed two studies employing R-BD R-βHB KME ingestion as a fuelling strategy alongside best practice in CHO-based fuelling; the first investigating intermittent running performance in field-based team sport athletes [49], and the second investigating 10-km running performance in middle and long distance runners [54]. In the first study, a 75-min running protocol that simulated match activity in soccer followed by a shuttle run to exhaustion was the model employed. Ingestion of 750 mg.kg^−1^ of R-BD R-βHB KME ingested as three boluses (50:25:25) at 20 min prior to exercise, and at 15 min and 45 min during exercise increased plasma [R-βHB] to > 1.5 mM at start of exercise, and reached ~ 2.6 mM at the end of exercise. However, R-BD R-βHB KME ingestion had no effect on 15-m sprint times during the 75 min period, nor did it improve subsequent shuttle run time to exhaustion (KME + CHO, 229 ± 72 s; CHO, 267 ± 96 s) [49]. Our second study was analogous to Cox et al. [44] by using endurance athletes, a 60-min pre-load (~ 65% $\dot{V}{\text{O}}_{{2{\text{peak}}}}$), and a TT performance test (10 km treadmill-based), albeit this was performed in runners and in the fed state [54]. Ingestion of 573 mg.kg^−1^ of R-BD R-βHB KME ingested as three boluses (50:25:25) at 30 min prior to exercise, at 20 min during the pre-load, and immediately before the TT, increased plasma [R-βHB] to ~ 1.0 to ~ 1.5 mM throughout exercise. However, R-BD R-βHB KME co-ingestion with CHO had no effect on 10-km TT performance compared with CHO alone (KME + CHO: 2402 ± 237 s; CHO: 2422 ± 246 s). Around the same time, Dearlove et al. [58] also observed no effect of R-BD R-βHB KME on exercise performance measured as *W*_max_ during a ~ 20-min incremental cycling exercise test in endurance-trained athletes (KME: 393 ± 22 W; water control, 389 ± 20 W). The single bolus of 330 mg.kg^−1^ of R-BD R-βHB KME in the fasted state increased whole blood [R-βHB] to 3.7 ± 0.3 mM at the start of exercise, which declined thereafter but remained > 2.0 mM throughout exercise [58].

The lack of performance benefit has been observed by another research group in a series of well-designed and well-executed studies [42, 70, 72], the most recent of which in fact observed a performance decrement associated with acute R-BD R-βHB KME [72]. The first two studies employed a design in which trained cyclists completed a simulated cycling race consisting of 3 h of intermittent intensity cycling exercise, a 15-min cycling TT, and a maximal sprint at 175% of lactate threshold (LT) [42, 70]. The dose of R-BD R-βHB KME was also identical in both studies, specifically 65 g of R-BD R-βHB KME ingested as 25 g, 20 g, and 20 g boluses at 60 min prior to, 20 min prior to, and 30 min into, the intermittent intensity cycling period. The aim of this dosing strategy was to increase circulating [R-βHB] to ~ 2–5 mM during the early stages of the trial, but to have returned to close to resting concentrations before commencing the 15-min TT and maximal sprint. Thus, circulating [R-βHB] was ~ 2–3 mM for the first 2 h of the intermittent intensity cycling period in both studies, and at the start of the TT averaged 0.5 ± 0.5 mM [42] and 0.8 ± 0.4 mM [70]. However, neither study observed a difference between R-BD R-βHB KME and control conditions for performance in either the 15-min TT or maximal sprint [42, 70] (Table 2).

The same group then investigated the effects of R-BD R-βHB KME on performance in a 30- minute cycling TT and a maximal sprint at 175% LT [72]. The performance tests were preceded by a 60-min pre-load of intermittent intensity cycling exercise during which 50 g of R-BD R-βHB KME was ingested as 25 g each at 30 min and 55 min into the pre-load period, and resulted in an increase in circulating [R-βHB] to a stable ~ 3.5 mM throughout the TT. Although performance in the maximal sprint was not impacted, performance in the 30-min TT was impaired by ~ 1.5% (95% CI − 0.2% to − 2.6%) after ingestion of R-BD R-βHB KME [72]. More recently, the ingestion of R-BD R-βHB KME (600 mg.kg^−1^ as a single bolus 35 min prior to exercise) was investigated for effects on performance in a 3 kJ.kg^−1^ cycling TT that had been preceded by a 30-min pre-load at VT [38]. Plasma [R-βHB] increased to ~ 3.9 M at commencement of the pre-load, and remained at ~ 3.5 mM at the start of the TT, but in these endurance-trained male and female athletes, similar performance outcomes were observed between conditions (CHO + KME: 16:25 ± 2:50 min:s, CHO + PLA: 16:06 ± 2:40 min:s) [38].

In contrast to these studies failing to observe an ergogenic effect, one recent study has observed a performance benefit in professional rugby union players performing intermittent exercise in a simulated rugby union-specific match-play protocol [86]. Compared with CHO alone, ingestion of 590 mg.kg^−1^ of R-BD R-βHB KME with CHO before and during exercise produced whole blood [R-βHB] of > 2.0 mM, and resulted in a 0.33 ± 0.41 s (2.1%) improvement in the average time to complete a sustained high-intensity performance test performed regularly throughout the protocol. However, 15-m sprint times and sled push performance during the protocol were not different between conditions [86]. Another report of the ergogenic effect of R-BD R-βHB KME ingestion is in response to 10 days of daily supplementation with R-BD R-βHB KME encompassing 6 days of a simulated endurance cycling race [87]. The pre- and post-intervention performance tests consisted of 90 min of cycling at 70% $\dot{V}{\text{O}}_{2\max }$ followed by an incremental test to exhaustion (~ 20 min), and these same tests were performed each day as the race simulation. In a parallel-group design, there was no difference in performance from pre- to post-intervention when the R-BD R-βHB KME supplemented group was compared with a control group consuming a CHO-rich diet with placebo supplementation [87]. However, there was a benefit of R-BD R-βHB KME on 50% of race days as a result of ~ 6 to ~ 8% improvements in time to exhaustion on days 1, 4, and 6 of racing. A potential confounder is that these improvements are reported relative to the pre-intervention test, which was performed in the fasted state, whereas the race days were performed in the fed state and the drinks provided were co-ingestion of CHO and KME (196.5 mg.kg^−1^ of R-BD R-βHB KME immediately before and after 60 min of exercise) producing whole blood [R-βHB] of ~ 1.0 to ~ 2.0 mM. That said, in the CHO-supplemented group, a performance improvement was only observed on day 1 of racing, but the small sample sizes (*n* = 7 per group) and parallel group design are methodological issues that preclude a firm conclusion about the ergogenic effect observed.

Most of the studies described above have been included in recent meta-analyses that unsurprisingly conclude that at present there is a lack of evidence for the ergogenic effect of ketone esters [91, 96, 224], yet in endurance sports such as professional cycling, the use of ketone esters reportedly remains widespread [225–227]. The divergent findings in relation to performance between those subsequent studies and the original finding of an ergogenic effect [44] could potentially be explained by a variety of factors including the specifics of the performance tests, the training level of the participants, the fed/fasted status and nutrient co-ingestion prior to and during exercise and the performance tests, the presence of GI disturbances, or the circulating [R-βHB] reached during exercise (Fig. 1). Therefore, the possibility remains that there are specific athletic contexts where acute ingestion of ketone esters would provide an ergogenic effect, but it is remarkable how little support there is for this benefit given the number of studies both with diverse designs, and from independent research groups.

## Effects of Ingestion of the R-BD R-βHB Ketone Monoester on Recovery From Exercise

Following intense exercise, the stimulation of glycogen resynthesis and muscle protein synthesis (MPS) via appropriate nutrition strategies is often given high importance as part of the recovery process [228, 229]. Athletes are recommended to follow optimal CHO and protein-based fuelling related to the type, timing, and amount of CHO and protein to facilitate this recovery process and prepare the next session of exercise or performance. Ingestion of CHO at a rate of 1.2 g.kg^−1^.h^−1^ during the first 4 h of recovery maximises the rate of muscle glycogen resynthesis; the addition of 0.4 g.kg^−1^.h^−1^ protein added to ‘suboptimal’ CHO doses during recovery can increase the rate of muscle glycogen resynthesis compared with suboptimal CHO alone [228]. When the time between exercise bouts is limited (< 8 h), nutritional strategies to augment glycogen resynthesis for subsequent exercise performance are often considered a priority [229].

As long ago as the 1930s, a post-exercise rise in *urinary* KBs was observed and then named the ‘Courtice-Douglas effect’ [230], which suggested that KBs had some role in metabolic responses during and/or after prolonged exercise. During recovery from prolonged aerobic exercise, a pattern of post-exercise ketosis (PEK) is often observed for several hours as circulating [KB] in the range of ~ 0.3 to ~ 2.0 mM [231]. Similar effects have also been observed after resistance exercise [232, 233]. The magnitude and time course of PEK are largely dependent on nutrient intake, that is, attenuated by high CHO feeding and alanine ingestion, and augmented by CHO restriction [181, 234–239]. During the post-exercise recovery period, in contrast to the reliance on CHO metabolism during exercise, muscle glycogen resynthesis has a high metabolic priority and is facilitated by an increase in fat oxidation and sparing of CHO sources for energy provision [240–243]. The priority for muscle glycogen resynthesis is observed even during CHO restriction [242, 244–246], and is achieved through non-CHO sources such as lactate and alanine being used for hepatic gluconeogenesis and redistribution to skeletal muscle [247]. In a post-absorptive state, over 90% of gluconeogenesis is accounted for by gluconeogenic amino acids (alanine, glutamine), lactate, and glycerol [248], due to the increase in gluconeogenic substrate availability to the liver and for preserving circulating [glucose]. The physiological role of PEK may be to contribute to repletion of muscle and liver glycogen given the ability of KBs to inhibit glycolysis and increase the conversion of glucose to glycogen [198, 199]. In addition to repletion of muscle glycogen, KBs have anabolic and anti-catabolic effects in skeletal muscle [136, 138] (described further in Sect. 10.4), including the observation of a 10% increase in MPS during 6-h infusion of sodium R,S-βHB to achieve circulating [R-βHB] of ~ 2 mM [138]. Together, these data suggest that acute ingestion of EKS could play a role in enhancing recovery after exercise by complementing existing best-practice nutrition strategies via augmenting muscle glycogen resynthesis and MPS (Fig. 1), but this contention has been subject to only a few studies to date.

The first study to investigate the role of EKS on recovery from exercise provided 573 mg.kg^−1^ of R-BD R-βHB KME immediately after a glycogen-depleting bout of exercise (115 ± 2 min) alongside a 2-h 10 mM hyperglycaemic clamp [48]. The 10 mM hyperglycaemic clamp was chosen in order to increase the circulating [glucose] to a concentration of at least what is evident during optimal post-exercise CHO feeding, although the authors did note that the 10 mM concentration was ‘supraphysiological’ [48]. Studies of CHO alone or CHO-protein co-ingestion after glycogen-depleting exercise rarely observe circulating [glucose] of > 8 mM during the feeding period [47, 249–251]. A further caveat is that the taste-matched control condition was non-caloric, as compared with ~ 5 kcal.g^−1^ provided by R-BD R-βHB KME. Whole blood [R-βHB] peaked at 5.3 ± 0.5 mM and remaining increased to 3.3 ± 0.2 mM at the end of the clamp period. R-BD R-βHB KME ingestion was reported as increasing muscle glucose uptake by 32%, circulating [insulin] twofold, and resulting in 50% higher muscle glycogen concentrations compared to CON [48]. However, the stated 50% higher muscle glycogen concentration in the R-BD R-βHB KME condition represents the difference between absolute values of muscle glycogen concentration after the clamp (KME, 246 ± 32 mM.kg dw^−1^ vs. CON, 164 ± 13 mM.kg dw^−1^), regardless of immediate post-exercise muscle glycogen concentrations, which were lower in the CON condition (94 ± 15 mM.kg dw^−1^ ) than in KME (132 ± 20 mM.kg dw^−1^). In relative terms, muscle glycogen concentrations at the end of the 2-h recovery period increased by 86% (114 ± 23 mM.kg dw^−1^) with KME, and by 74% (70 ± 13 mM.kg dw^−1^) with CON [48]. Therefore, the difference between conditions as relative change in muscle glycogen concentration was modest. The mechanism for the augmented muscle glycogen resynthesis was proposed as the doubling of circulating [insulin] during the KME condition (KME: 31 ± 5 mU.L^−1^; CON: 16 ± 3 mU.L^−1^), but early post-exercise muscle glycogen resynthesis may in fact be independent of circulating [insulin] when muscle glycogen concentrations are initially depleted below 150–200 mM.kg dw^−1^ [228]. However, a subsequent study in mice does support the effect of R-βHB to enhance early post-exercise glycogen resynthesis independent of insulin [252]. In that study, male mice swam for 60 min after which the epitrochlearis muscle was excised and treated ex vivo for 2 h with either glucose (8 mM), insulin (60 mU.L^−1^), or glucose and insulin plus 1, 2, or 4 mM of sodium R,S-βHB. Muscle glycogen concentration was unchanged after treatment with 1 mM sodium R,S-βHB, but was increased by ~ 15% and ~ 35% after treatment with 2 mM and 4 mM sodium R,S-βHB, respectively [252].

Conversely, in another study of human participants with post-exercise ingestion of R-BD R-βHB KME, there was no effect on muscle glycogen resynthesis during a 5-h recovery period [47]. That study employed a 90-min unilateral leg extension exercise protocol to deplete muscle glycogen (~ 60% to ~ 175 mM.kg dw^−1^) followed by a feeding strategy in the form of recovery drinks consumed hourly to provide 1.0 g.kg^−1^.h^−1^ CHO and 0.3 g.kg^−1^.h^−1^ whey protein hydrolysate. This feeding strategy was chosen to elicit maximal rates of MPS and muscle glycogen resynthesis during the post-exercise recovery period [229]. Participants also ingested 0.5 g.kg^−1^ of R-BD R-βHB KME immediately after exercise, followed by 0.25 g.kg^−1^.h^−1^ thereafter, which produced whole blood [R-βHB] in the range of ~ 3 to ~ 4 mM throughout the recovery period [47]. Compared with the control condition (isocaloric long-chain fatty acids), there was no difference in circulating [insulin] between conditions (~ 15 mU.L^−1^), but circulating [glucose] was lower by ~ 1 mM throughout the KME condition. Obvious differences in study design may explain the contrasting findings of these two human studies [47, 48], including the different exercise modalities and magnitude of glycogen depletion, the different methods of increasing glucose availability (i.e. nutrient provision), and the different durations of the recovery period. One notable secondary outcome in that study by Vandoorne et al. [47] was the observation with R-BD R-βHB KME ingestion of greater phosphorylation of S6K1 and 4E-BP1, which are two key regulators of MPS via the canonical mechanistic target of rapamycin (mTOR) pathway [253]. Moreover, in vitro experiments in C2C12 myotubes demonstrated that the combination of 1.4 mM AcAc and 4 mM βHB augments S6K1 and 4E-BP1 signalling in response to 1.5 mM leucine, coincident with an increase in MPS, compared with 1.5 mM leucine alone [47]. Together, these data are suggestive of the anabolic action of KBs in skeletal muscle, and may be fruitful avenues for research in several domains [90] (Sect. 10.4).

Recovery of muscle function after exercise-induced muscle damage (EIMD) through eccentric contraction-dominant exercise has been the subject of one study of supplementation with EKS [66]. Using a parallel group design, participants ingested either R-BD R-βHB KME (360 mg.kg^−1^ per dose), or isocaloric CHO, twice daily (morning and 30 min prior to sleep) for 2 days after performing an exercise session consisting of 5 × 20 repetitions of 60-cm drop jumps. Supplementation commenced ~ 15 min after completion of the session, with acute ingestion producing whole blood [R-βHB] of ~ 4.0 and ~ 4.1 mM at 30 and 60 min, respectively, after ingestion before a decline to ~ 1.3 mM at 180 min. However, between-group differences were not evident at + 24 or + 48 h of recovery for muscle function (maximum voluntary isometric contraction and countermovement jump), markers of muscle damage and inflammation, or muscle soreness [66]. EIMD involves a complex interplay of many mechanisms that ultimately manifest as changes in muscle function and circulating factors over several days, and can range in severity depending on the exercise protocol used [254]. Hence, the rationale for this line of investigation is that KBs have pleiotropic roles in cellular metabolism [5, 255], and of relevance to recovery from muscle damage are effects on inflammation, oxidative stress, and skeletal muscle satellite cell activation [27, 112, 139, 256–258]. Nutrition interventions can, in principle, ameliorate EIMD and associated outcomes [254], and given the plausible mechanisms by which KBs may impact these processes, further studies are warranted to investigate the potential effects of EKS on recovery from EIMD.

Somewhat related to the concept of recovery is that of overreaching and overtraining. One study has investigated the effect of daily consumption of R-BD R-βHB KME (75 g.d^−1^ as 3 × 25 g) during 3 weeks of overreaching [99]. This study, in physically active young men, involved an intensive cycling-based training programme incorporating constant-load aerobic exercise training, intermittent endurance training, and high-intensity intermittent exercise training, often twice daily for 6 days of each week. R-BD R-βHB KME was added to a CHO- and protein-based recovery drink consumed after each training session, and an additional 25 g of R-BD R-βHB KME was consumed prior to bedtime. Ingestion of isocaloric MCTs (16.4 g at each time point) was used for the control condition. One performance measure was average power output during a 30-min TT and subsequent 90-s sprint, performed at baseline, day 7, day 14, and day 21 of training, and at day + 3, day + 7 after training ceased, but there were no between-condition differences in these measures. However, power output during a 30-min cycling TT performed after a 90-min pre-load was ~ 15% greater with R-BD R-βHB KME compared with control on day 18 of the training programme (KME: 216 ± 8 W; CON: 188 ± 14 W), whereas a range of other indicators of overreaching were attenuated including nocturnal adrenaline, and resting, submaximal, and maximal heart rate responses [99]. Together, these results suggested that daily consumption of R-BD R-βHB KME blunted the symptoms of overreaching in this cohort. While this study was well performed and clearly an enormous undertaking for participants and researchers, these finding have been the subject of much discourse [259–266]. In particular, a focus has been on the dietary control around the performance tests, and important between-group differences that arose in the dietary intake data. A ‘spontaneous’ ~ 20% increase in energy intake was observed in the KME group, which was attributed to a greater increase in serum [GDF15] compared with the control group, with no differences between groups in plasma [leptin] and [ghrelin]. However, dietary intake data were collected for only 2 d.wk^−1^ during the training period and may not be a complete representation of the participants’ ad libitum weekly intake. Furthermore, during week 1, dietary data were collected on testing days − 4 and − 3; during weeks 2 and 3, dietary data were collected on testing days − 2 and − 1. Testing day − 1 included a prescribed CHO-rich meal for both groups, so while not discounting the difference in energy intake observed, the participants’ ‘free living’ energy intake was not being definitively captured. In support of an effect of daily consumption of R-BD R-βHB KME altering dietary intake is a subsequent study simulating a 6-day endurance cycling race [87]. Compared with participant intakes on a CHO-rich control diet that were largely similar between rest days and race days, average daily energy intake increased by ~ 700 kcal during the 6-day race period in the R-BD R-βHB KME-supplemented (1179 mg.kg^−1^; ~ 11% of energy intake) group [87].

Overall, the data on recovery and overreaching are preliminary, but suggest that there may be efficacy for EKS in these contexts. Replication of the effect of daily R-BD R-βHB KME ingestion to blunt the effects of overreaching would be welcome, whereas for acute recovery, employing ecologically valid approaches to glycogen depletion and the provision of CHO and protein after exercise will elucidate whether the addition of EKS confers any benefit to glycogen resynthesis and MPS in practical terms. Lastly, whether the addition of EKS can augment maximal rates of MPS induced by protein feeding after resistance exercise also warrants further investigation.

## Methodological Considerations and Future Directions

Applying research on performance enhancing dietary supplements poses a greater challenge than with other forms of dietary supplements due to the scarcity of quality studies applicable to the elite athlete [267]. A range of best-practice guidelines for conducting dietary supplement research in this cohort are now available [267, 268], and include features such as adequate sample sizes determined a priori, clearly defined and objectively measured inclusion criteria, double-blinding with randomisation to the control or experimental group or in a crossover design where participants receive both treatments, use of a verified non-contaminated substance with an appropriate protocol (dosage and timing), standardised pre-trial preparation across trials (exercise, diet, caffeine, alcohol ingestion), environmental factors during each trial (temperature, encouragement) that should also mimic conditions of real-life events (ecological validity), and the use of a reliable and valid performance test to assess the primary outcome including the assessment of order effects. In relation to this is the recent development of comprehensive guidelines and standards of reporting in sports nutrition and exercise metabolism (PRESENT, Proper Reporting of Evidence in Sport and Exercise Nutrition Trials) [269], and the suggestion that when designing a study or writing a manuscript, authors may find it useful to quantify the translational potential of their research, highlighting the strengths of their respective designs [270]. Like other domains in exercise science [271], females have been underrepresented in EKS studies to date, and where females have been included [21, 36, 38, 44, 54, 58–61, 66, 67, 78, 84, 87], between-sex differences have not been powered for nor explored statistically, but warrant further investigation given the between-sex differences in substrate utilisation and exercise performance [272]. Other methodological considerations and future directions specific to research with EKS are outlined in the following subsections.

### Measurement of [R-βHB]

As described in Sect. 2, measures on whole blood capillary samples using POC devices may overestimate [R-βHB] by as much as ~ 0.5 mM [34, 35, 38, 113, 114, 116, 121, 123]. While POC devices are convenient, direct laboratory assay or ELISA assay should be considered the criterion method for measurement of plasma/serum [R-βHB] in order to standardise the reporting and interpretation of results, especially when [R-βHB] is in the range of mild ketosis. If using POC measures, the possibility of overestimation should be acknowledged, especially in the low concentration range (e.g. [R-βHB] < 1.0 mM), and data reported as *whole blood* [R-βHB]. The use of POC measures also raises an issue for the adequate blinding of researchers in a performance study, as this method provides an immediate indication of an increase in whole blood [R-βHB], which is otherwise negligible in placebo or control conditions, especially with ingestion of CHO. In such scenarios, blinding can be maintained by having an independent researcher who is not involved in the performance testing analyse samples for [R-βHB] [66, 72].

For assays of plasma/serum [AcAc] and [R-βHB], the handling and storage of samples becomes an important consideration. R-βHB is a very stable metabolite in plasma and whole blood samples, allowing for measurement up to 7 days after sample collection when stored at 4 °C, and for substantially longer periods when stored at − 80 °C [273]. In contrast, the degradation of AcAc in plasma samples may occur such that guidelines suggested that measurement must take place within 24 h of sample collection, or be stored for no longer than 3 days at − 20 °C (i.e. 80% and 30% of AcAc was lost from plasma samples after 3 days of storage at room temperature and − 20 °C, respectively) [274, 275]. However, degradation of AcAc in plasma is reduced when stored at − 80 °C (i.e. only 14% of AcAc lost over 40 days of storage) [276]. Moreover, AcAc is stable when de-proteinised with perchloric acid and stored at − 80 °C for 60 days, and is more stable in plasma than whole blood samples, because de-proteinisation removes BDH activity so that AcAc is not being reduced to βHB [277].

[AcAc] has rarely been measured in the studies of EKS ingestion cited in this review, but is elevated as expected after acute ingestion of R,S-BD AcAc KDE [35], and, less expectedly, after acute ingestion of R-BD R-βHB KME [34, 38]. In the latter studies, the ratio of [R-βHB]:[AcAc] was observed to be ~ 6:1 or ~ 4:1 when R-BD R-βHB KME was ingested in the fasted or fed state, respectively [34], whereas after 30 min of moderate intensity exercise commenced in the fed state, this ratio was ~ 1.7:1 [38]. In that study [38], [R-βHB] had declined from the onset of exercise, presumably reflecting an increase in R-βHB disposal during exercise [31], whereas [AcAc] continued to increase during exercise [38]. The physiological significance of an increase in [AcAc] in this manner remains to be established because the primary focus has been on [R-βHB] in studies to date. As reviewed elsewhere [4, 5, 255], there are cellular signalling and metabolic effects common to AcAc and R-βHB, but there are also several intriguing differences between the molecules that may be avenues for future research exploring the performance and therapeutic potential of the various forms of EKS.

### Type, Dose, and Timing of Exogenous Ketone Supplements (EKS)

One issue with the studies of EKS ingestion to date is that supplement doses are reported in a variety of units such as mg.kg^−1^ [35, 36, 38, 44, 49, 54, 56–60, 62, 67, 68, 78, 81, 86, 87], mM.kg^−1^ [34], kcal.kg^−1^ [53], mL.kg^−1^ [48, 50], g.kg^−1^.h^−1^ [47], and absolute value in g [21, 37, 42, 61, 64, 71, 72, 82–85, 99, 107]. In the case of βHB salts, whether they are racemic or non-racemic is often not stated, nor is it always clear whether the dose refers to the gram value of R-βHB, R,S-βHB, or the supplement serving itself. Researchers and practitioners require a standardised reporting method to inform their own work and avoid misinterpretations. Standardised reporting to include both the mg.kg^−1^ of the supplement itself, and mg.kg^−1^ βHB and/or AcAc (based on the % purity of the product) is recommended if such data are available. Similarly, a purity analysis to define the R-βHB and S-βHB components should be undertaken for non-racemic βHB salts. These approaches may serve to better inform practices aimed at avoiding GI symptoms, and achieving circulating [KB] in the desired concentration range and time frame before, during, and after exercise, depending on the aim of the intervention.

### Utility of EKS in Low Oxygen States

The reduced cellular oxygen availability characteristic of hypoxia attenuates cellular mitochondrial respiratory capacity, systemic blood volume, blood buffering capacity, and blood flow to working muscle, while augmenting inflammation, oxidative stress, heart rate, and respiratory rate [278–280]. These cellular and systemic responses can induce acute mountain sickness, sleep disturbances, cognitive impairment, and reduce physical performance [281–284]. Acclimation to altitude exposure includes increased red blood cell production, blood volume, and muscle buffering capacity [285–287], but these adaptations typically require several weeks of exposure to manifest. Therefore, a range of strategies have been employed to mitigate these acute adverse effects such as altitude descension, acetazolamide, erythropoietin, intravenous iron infusion, and sildenafil [285, 287–290]. However, these each have limited effectiveness or present notable side effects: acute altitude descension may not be logistically feasible; acetazolamide has been demonstrated to reduce both cognitive and physical performance, accrue side effects, and does not increase muscle oxygenation [285, 287]; erythropoietin may only provide ergogenic effects up to 3500 m [289]; intravenous iron infusion may not be feasible for all individuals [290]; and sildenafil can exaggerate symptoms of mountain sickness including headaches [288]. An increased reliance on, or preference for, CHO for energy provision in hypoxic environments such as moderate-to-high altitude is hypothesised [291], but CHO ingestion does not reliably augment performance at moderate-to-high altitude [292–294]. In fact, aerobic exercise performed during acute high altitude exposure elicited lower exogenous glucose oxidation, glucose turnover, and glucose disposal, while concomitant increases in circulating [glucose] and [insulin] suggested a reduced sensitivity of skeletal muscle to glucose uptake in hypoxia compared with exercise in normoxia [295].

Taken together, alternative fuelling strategies that attenuate declines in systemic and skeletal muscle oxygenation, exercise performance, and symptoms of mountain sickness may provide ergogenic benefit during moderate-to-high altitude exposure. Acute ingestion of R-BD R-βHB KME can increase systemic and skeletal muscle oxygenation at rest and during exercise with hypoxic exposure [71, 76]. During exercise, this effect was not associated with either positive or negative effects on endurance performance, whereas at rest, the decline in cognitive performance during hypoxic exposure (simulating an altitude of ~ 5000 m) was attenuated by acute nutritional ketosis [76]. Acute ingestion of R-BD R-βHB KME produces a mild acidosis [34, 42, 69–72] in a similar manner to the induction of mild acidosis with acetazolamide, which coupled to the effect of bicarbonate to attenuate the improvement in oxygenation after R-BD R-βHB KME ingestion [71], suggests that any oxygen advantage at altitude with acute nutritional ketosis is likely to be driven in part by the mild metabolic acidosis. Future studies should continue to investigate the value of acute nutritional ketosis in mitigating the adverse effects of acute altitude exposure and other hypoxic insults including physical and cognitive performance outcomes, the overlapping or distinct roles of R-βHB and AcAc, and the mechanistic basis for any effects observed.

### Utility of EKS as Anabolic or Anti-Catabolic Compounds

KBs were first observed to be associated with sparing of body proteins during catabolic insults over half a century ago [296, 297], and several mechanistic studies have provided evidence for the anti-catabolic effects of KBs themselves [90]. These include observations of the effect of KBs to attenuate skeletal muscle catabolism in in-vitro myotubes [256, 298], pre-clinical cancer cachexia including pancreatic, colon, and metastatic cancers [298–300], human refractory cancer cachexia [301], endotoxin-mediated inflammatory stress [139, 302], and muscular dystrophy [256]. These anti-catabolic effects, coupled with the potential anabolic effects of acute nutritional ketosis described in Sect. 9, suggest that EKS may support maintenance of skeletal muscle mass in high stress environments [255], including where athletes are at risk for excessive protein breakdown, inadequate recovery, and overreaching [99]. Admittedly, most human studies on this theme have been acute in nature [47, 136, 138, 139, 302] and rely on the interpretation of acute responses as surrogate markers for longer term effects on skeletal muscle. Yet the relative ease with which acute nutritional ketosis can be achieved daily for extended periods of time with EKS [75, 99, 100, 106, 107] has expanded the potential utility of EKS in diverse settings for mitigation of skeletal muscle atrophy [90, 255]. Future studies should aim to extend the many promising acute observations into longer term investigations of the therapeutic efficacy of daily ingestion of EKS on skeletal muscle mass and function in response to diverse atrophy stimuli. Of particular relevance to athletes would be scenarios such as functional overreaching and intensified training, phases of intentional weight loss via energy restriction such as in weight category sports, and periods of forced inactivity due to injury.

### Utility of EKS in Traumatic Brain Injury (TBI)

TBI results from a rapid insult to the brain resulting in axonal shearing, vascular damage, and increased permeability of the blood–brain barrier [95, 303–305]. In the next minutes to year(s), reduced blood flow and oxygen availability, as well as an increase in carbon dioxide production, oedema, intracranial pressure, inflammation, oxidative stress, excitotoxicity, and degeneration, drive the pathological sequence to altered consciousness, seizures, cognitive decline, motor dysfunction, mental and behavioural disorder, and in some severe cases, death [95, 303–305]. Central to the pathogenesis of TBI and associated clinical outcomes is metabolic dysfunction, which is manifested as peripheral and brain insulin resistance, hyperglycaemia, and cerebral hyper- to hypometabolic transition, amongst other effects that have well recognised adverse effects on brain and systemic health associated with TBI morbidity and mortality [303, 306–310]. TBI can occur in athletic contexts, with the severity ranging from small but repeated sub-concussive insults up to overt high impact insult resulting in loss of consciousness [311, 312].

Ketosis achieved by ketogenic diet or ingestion of EKS has been widely hypothesised to present a promising prophylactic and/or post-injury therapeutic strategy for TBI through a mechanism whereby KBs serve as an alternative substrate for energy provision during the post-injury period of impaired glycolytic metabolism [40, 95, 255, 313–316]. In the post-injury period, several features of bioenergetic impairment are observed including increased flux of glucose through the pentose phosphate pathway, free radical production, activation of poly-ADP ribose polymerase via DNA damage, and inhibition of glyceraldehyde dehydrogenase via depletion of the cytosolic NAD^+^ pool. Ultimately, glucose becomes a less favourable energy substrate and ATP provision decreases, whereas KBs are an alternative substrate that can contribute significantly to cerebral metabolism [40, 95, 255, 313–316]. A recent pre-clinical study of TBI using controlled cortical impact in Sprague–Dawley rats demonstrated therapeutic promise for EKS as post-injury daily oral gavage of R-BD R-βHB KME protected against TBI-induced morphological and functional deficits [317]. Further pre-clinical studies and human trials are required to determine the optimal protocol to support cerebral ketone metabolism in the post-injury brain, and to validate the neuroprotective benefits of therapeutic ketosis in humans in both prophylactic and post-TBI applications.

## Conclusions

There is little doubt that AcAc and R-βHB have wide-ranging metabolic and molecular effects on organs such as the brain, heart, and skeletal muscle, and that the commercial availability of ingestible EKS has led to the recent interest in KBs in athletic contexts, and resurgent interest in KBs in therapeutic contexts. Despite the mechanistic bases for potential beneficial effects of EKS, the evidence at present is overwhelmingly against EKS being an ergogenic aid for athletic performance. Yet questions remain about whether there are optimal dosing strategies (especially using ketone esters), specific athletic populations, or specific exercise challenges in which acute ingestion of EKS may provide a performance benefit. Additionally, future research should investigate whether there are other athletic contexts where EKS are efficacious given the positive, albeit preliminary, data from studies on overreaching, acute hypoxic exposure, and traumatic brain injury.
